# Generation and UV-photolysis of *N*-methyleneformamide†

**DOI:** 10.1039/d5sc02777b

**Published:** 2025-05-15

**Authors:** Viktor Paczelt, Vladimir D. Drabkin, Daniel Kühn, André K. Eckhardt

**Affiliations:** a Lehrstuhl für Organische Chemie II, Ruhr-Universität Bochum 44801 Bochum Germany Andre.Eckhardt@ruhr-uni-bochum.de

## Abstract

Simple imines have been detected in space and are considered as building blocks to larger heteroaromatic, cyclic and biologically relevant compounds. Herein, we revisited the formation of the simplest acylimine, namely *N*-methyleneformamide, by high-vacuum flash pyrolysis (HVFP) as well as its spectroscopic characterization by cryogenic matrix isolation infrared (IR) and UV/Vis spectroscopy. *N*-Methyleneformamide prefers a *gauche* over an s-*trans* conformation in contrast to the parent 1,3-butadiene. In UV-photolysis experiments we identified formaldehyde:HCN and formaldimine:CO complexes as the major decomposition products. As further photolysis products we observed HCN:CO and HNC:CO complexes. All experimental findings are supported by deuterium labeling experiments and high-level *ab initio* coupled cluster calculations. *N*-Methyleneformamide should be considered as a candidate for an interstellar search. The sequence R_2_C=N–RC=O also occurs in cytosine making the title compound highly relevant for prebiotic chemistry and the search for the molecular origins of life.

## Abstract 2

*N*-Methyleneformamide is the simplest acylimine and was characterized under matrix isolation conditions at cryogenic temperatures by infrared and UV/Vis spectroscopy. The compound should be considered as a potential interstellar molecule.

## Graphical abstract

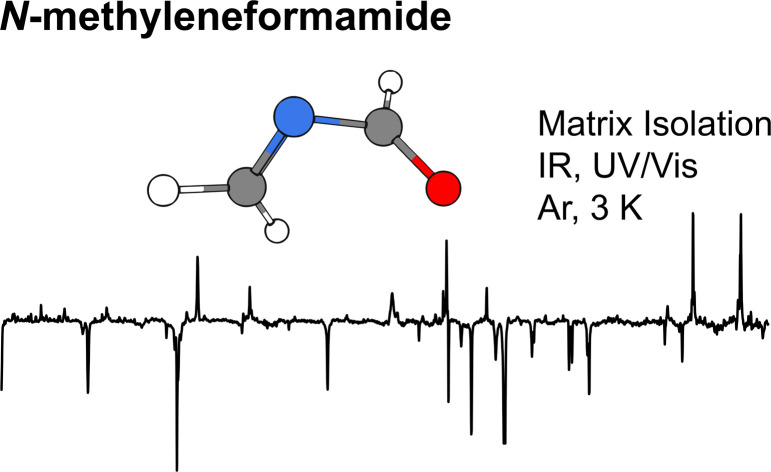

## Introduction

**Scheme 1:**
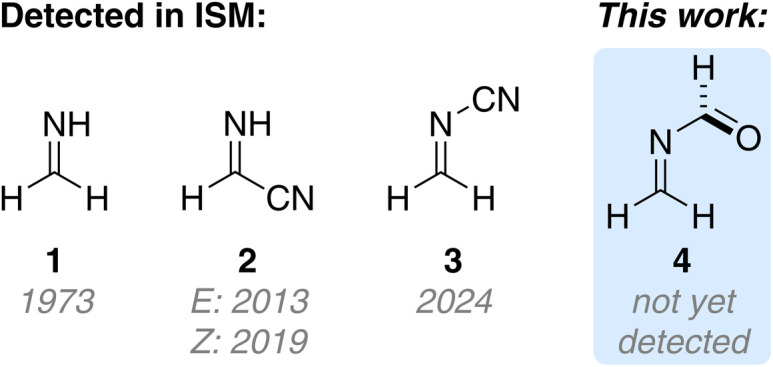
Formaldimine (1) and the HCN dimers cyanoformaldimine (2) and *N*-cyanomethanimine (3) have been detected in the interstellar medium (ISM) while *N*-methyleneformamide (4) is not yet detected.

Imines are fundamental building blocks in prebiotic chemistry, *e.g.*, in Strecker's amino acid synthesis or in HCN dimerization and the adenine forming Ferris Orgel reaction.^1–3^ Simple imines are continuously discovered in space, including formaldimine (H_2_C=NH, 1),^4^ cyanomethanimine (NCHC=NH, 2)^5^ and others (Scheme 1).^6–9^ Only last year the high energy isomer of the HCN dimer 2, namely *N*-cyanomethanimine (H_2_C=NCN, 3) including the prebiotically relevant NCN backbone, was discovered in space.^10^ This provides evidence that other differently substituted methanimines might also be present in space including *N*-methyleneformamide (4). Computational studies predict the formation of 4 from H_2_CO and HCN,^11^ as well as HCNO and CH_2_ under plausible interstellar conditions.^12^

*N*-Methyleneformamide is the simplest *N*-acylimine and was already part of theoretical^13–19^ and experimental investigations.^20^ It is noteworthy that the sequence R_2_C=N–RC=O also occurs in the nucleobase cytosine, making 4 highly relevant for the field of prebiotic chemistry. *N*-Methyleneformamide was previously generated from a cyclopentadiene based precursor in a retro-aza-Diels–Alder reaction at 450 °C in the gas phase under high vacuum and characterized using low-temperature infrared spectroscopy.^20^ However, only the two most prominent vibrations, namely the CO and CN stretching vibrations at 1735 and 1695 cm^−1^, were observed in a solid film. Even in the condensed phase and in the absence of a solvent, 4 showed propensity to polymerize at −196 °C. Due to the high reactivity, a low temperature nuclear magnetic resonance (NMR) spectrum could not be reported in contrast to other reactive *N*-acylmethanimines.^20^ In a mass spectrometric and microwave spectroscopic study only *trans*-nitrosoethylene (H_2_CHCNO) could be identified as the elusive C_2_H_3_NO isomer.^21^ To the best of our knowledge the microwave spectrum of 4 has not yet been reported, despite its high dipole moment of 3.0 D (*gauche*, B3LYP/6-311+G**).^18^

*N*-Methyleneformamide is a 1,3-butadiene heteroanalog, preferring a *gauche* over a planar s-*trans* conformation according to density functional theory (DFT) calculations (*cf.* Computational analysis, Fig. 5).^18,19^ The presence of both an imino and a carbonyl functional group gives 4 a rotational profile around the C=N–C=O dihedral angle, which is at odds with other (hetero)butadiene analogs.^22^ The energetic preference for the *gauche* conformer was rationalized with electronic repulsion effects.^11^ The repulsive interactions are greater than the conjugation effect in the two planar conformations of 4.^18,19^ Only recently, the non-planar *gauche* preference for the parent 1,3-butadiene was demonstrated experimentally in a Fourier-transform microwave (FTMW) spectroscopy study.^23^ The lower energy s-*trans* conformer does not possess a dipole moment and is invisible to radio astronomy. However, the cyano tagged butadiene analog, namely s-*trans-E-*1-cyano-1,3-butadiene, was observed in TMC-1 in 2023 suggesting that more heteroatom substituted butadienes might be present in space.^24^

Panda *et al.* explored various C_2_H_3_NO isomers in the context of interstellar chemistry. Methylisocyanate (H_3_CNCO), glycolonitrile (HOCH_2_CN), 4 and 2-iminoacetaldehyde (HC(O)CHNH) were identified as the lowest energy C_2_H_3_NO isomers.^11^ We reported recently the spectroscopic characterization of interstellar relevant imines by UV photolysis of the corresponding azide precursors.^25–27^ In the UV photolysis of 2-azidoacetaldehyde we identified 2-iminoacetaldehyde and two unassigned bands at 993.7 and 1469.9 cm^−1^ that did not show any photochemistry. As 4 was suggested as a viable photoproduct but IR spectroscopically only characterized by two bands, we revisited the preparation of 4 and investigated its photochemistry experimentally under matrix isolation conditions.

## Results and discussion

### Infrared spectroscopy

**Scheme 2:**
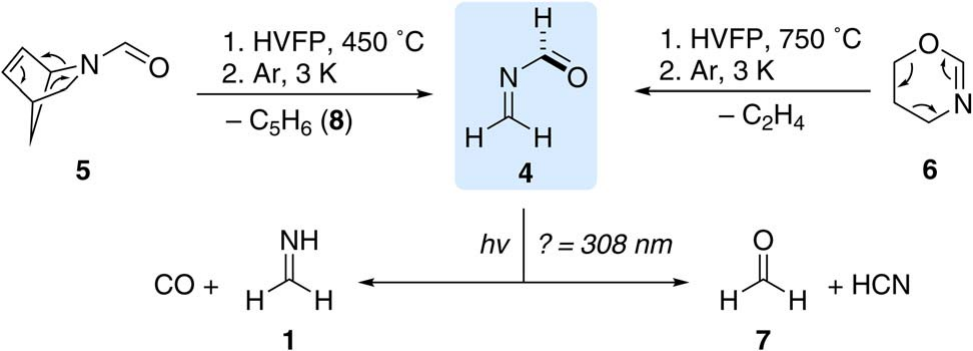
Gas phase generation of *N*-methyleneformamide (4) by high-vacuum flash pyrolysis (HVFP) of *N*-formyl-2-azabicyclo[2.2.1]hept-5-ene (5) and 5,6-dihydro-4*H*-1,3-oxazine (6) and subsequent trapping of all pyrolysis products in solid argon at 3.4 K. UV irradiation (*λ* ≤ 308 nm) of the matrix leads to the decomposition of 4 into formaldimine (1) and CO as well as formaldehyde (7) and HCN.

For the generation of 4 we synthesized *N*-formyl-2-azabicyclo[2.2.1]hept-5-ene (5) as described by Lasne *et al.* including the 3,3-deuterium isotopically labelled compound (Scheme S1†).^20^ We performed high-vacuum flash pyrolysis (HVFP) experiments with 5 at 450 °C and directly trapped all pyrolysis products with an excess of argon on a cold matrix window at 3.4 K (Scheme 2). Besides the well-known signals of matrix isolated cyclopentadiene (8) in argon,^28^ we identified another set of signals at 2956.8, 2908.0, 2873.7, 1719.4, 1664.0, 1473.1, 1372.6, 1196.1, 1057.0, 1030.5, 992.6, and 786.6 cm^−1^ (Tables 1 and S1† (extended version)), which we assigned to the fundamentals of *gauche*-4 based on our harmonic frequency calculations of *gauche*-4 at the CCSD(T)/cc-pVTZ^29–33^ and anharmonic frequency calculations at the B2PLYP/6-311++G(2d,2p)^34–36^ level of theory (Fig. S1, Table 1, S1†). It is worth noting that our experimental results are in very good agreement with the anharmonic calculations of the B2PLYP double hybrid functional as already pointed out in benchmark studies.^37^ There is no spectroscopic evidence for s-*trans*-4 in our pyrolysis spectrum. Cyclopentadiene and *gauche*-4 are the retro-aza-Diels–Alder reaction products of 5. We irradiated the matrix with UV light (*λ* = 254 nm, 40 min) and observed a 40% decomposition of *gauche*-4 along with the reported disrotatory photoinduced intramolecular cycloaddition of 8 forming bicyclo[2.1.0]pent-2-ene (BCP) as observed by its two most intense bands at 774 and 720 cm^−1^**(**Fig. S2†).^28^ We identified complexed formaldimine^38^ and carbon monoxide as well as the reported HCN:formaldehyde complex^39^ as the primary photolysis products of *gauche*-4 (Fig. S2†).

**Table 1:** Comparison of experimental fundamental vibrational frequencies of *gauche*-4 isolated in Ar at 3.4 K and calculated fundamental vibrational frequencies^a^

Mode	Approx. description	B2PLYP/6-311++G(2d,2p)		Experiment	
		*ṽ*_anh._/cm^−1^	*I*_anh._/km mol^−1^	*ṽ*_e*x*p._/cm^−1^	*I*_rel_
3	*δ*(NCO), *τ*(HCNC)	502.9	7.0	n.o	—
4	*τ*(HCNC)	790.5	8.6	786.6	w
5	*v*(CN), *δ*(NCO), *τ*(NCHO)	985.0	64.0	992.6	s
6	*τ*(NCHO)	1040.6	46.1	1030.5	m
7	*τ*(NCHH)	1078.5	17.3	1057.0	m
8	*δ*(HCN)	1207.1	18.6	1196.1	m
9	*δ*(HCO)	1376.8	7.7	1372.6	w
10	*δ*(HCH)	1488.9	15.6	1473.1	m
11	*v*(CN)	1665.6	127.8	1664.0	s
12	*v*(CO)	1712.1	163.3	1719.4	vs
13	*v*(CH)	2858.0	63.0	2874.7	m
14	*v*_sym_(CH_2_)	2912.4	21.5	2908.0	w
15	*v*_as_(CH_2_)	3045.6	15.5	3029.9	w

a rel. experimental intensities (w = weak, m = medium, s = strong, vs = very strong); n.o.: not observed.

**Fig. 1:**
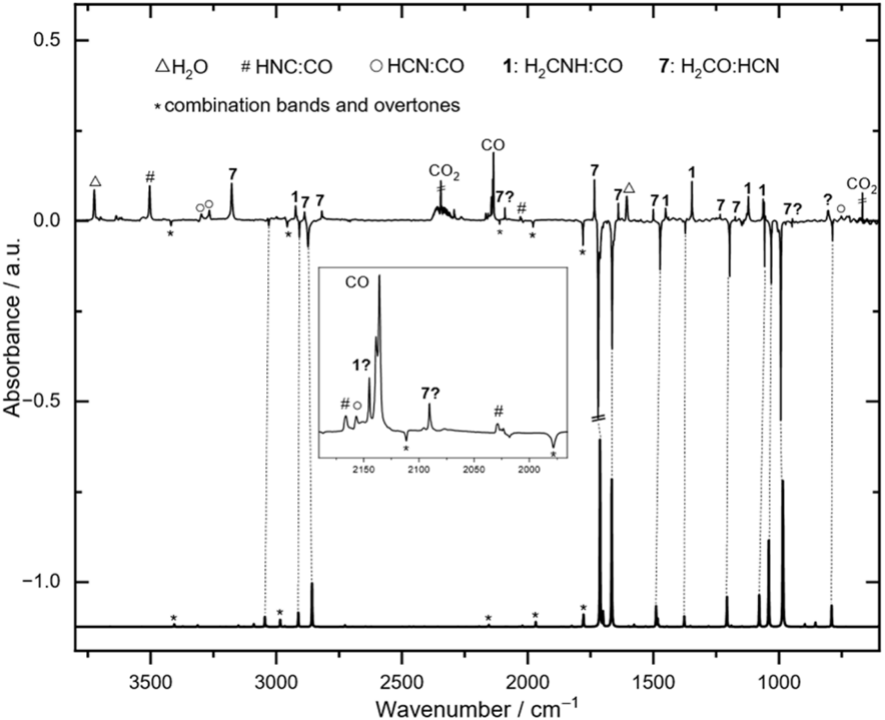
Top: Experimental IR difference spectrum between the matrix IR spectrum at 3.4 K recorded after 84 min pyrolysis of 6 at 750 °C and irradiation of the matrix with UV light (*λ* = 308–254 nm) for 270 min. Bottom: Calculated anharmonic IR spectrum of *gauche*-4 at the B2PLYP/6-311++G(2d,2p) level of theory. After UV irradiation *gauche*-4 decomposes into formaldimine:CO (1) and HCN:formaldehyde (7) complexes, which presumably further decompose into dihydrogen (IR inactive) and HCN:CO (○) and HNC:CO (#) complexes.

As we observed critical overlaps in the IR spectrum of *gauche*-4 with 8 and its photochemistry, we investigated another precursor for the generation of *gauche*-4. In the [4 + 2] retro-aza-Diels–Alder reaction of 5, cyclopentadiene acts as the diene and 4 as the dienophile. We synthesized 5,6-dihydro-4*H*-1,3-oxazine (6) as an alternative precursor (Scheme S2†). In the corresponding [4 + 2] retro-aza-Diels–Alder reaction ethylene and 4 should be the reaction products as the dienophile and diene, respectively. The oxazine heterocycle is highly sensitive and polymerizes as a neat liquid even at −26 °C in the dark within three days. We conducted HVFP experiments with 6 in the 450–750 °C temperature range (Scheme 2). The compound is more stable than 5 and starts decomposing at around 650 °C. At 750 °C we identified the same set of signals for *gauche*-4 as described in the pyrolysis of 5 as the major pyrolysis product along with the characteristic sets of signals for ethylene,^40^ HCN, formaldehyde, formaldimine, CO and undecomposed starting material (Fig. S3†). In contrast to 8, ethylene and the other pyrolysis side products do not show any photochemistry after UV irradiation (*λ* > 254 nm), and we observed a difference spectrum after photolysis of *gauche*-4 only (Fig. 1). The tiny signals in Fig. 1, which are pointing downwards and are marked with an asterisk (*) at 3419.0, 2984.4, 2111.6, and 1978.3 cm^−1^, are determined to be the corresponding overtone signals of the fundamentals of *gauche*-4 at 1719.4, 1473.1, 1057.0, and 992.6 cm^−1^. The characteristic signal in the carbonyl region at 1779.2 cm^−1^ is a combinational band of the fundamentals at 992.6 and 786.6 cm^−1^. After prolonged photolysis we also identified the HCN:CO and HNC:CO complexes as further photolysis products.^41^ We assume that IR inactive dihydrogen forms together with these complexes.

### Deuterium labelling experiment

**Fig. 2:**
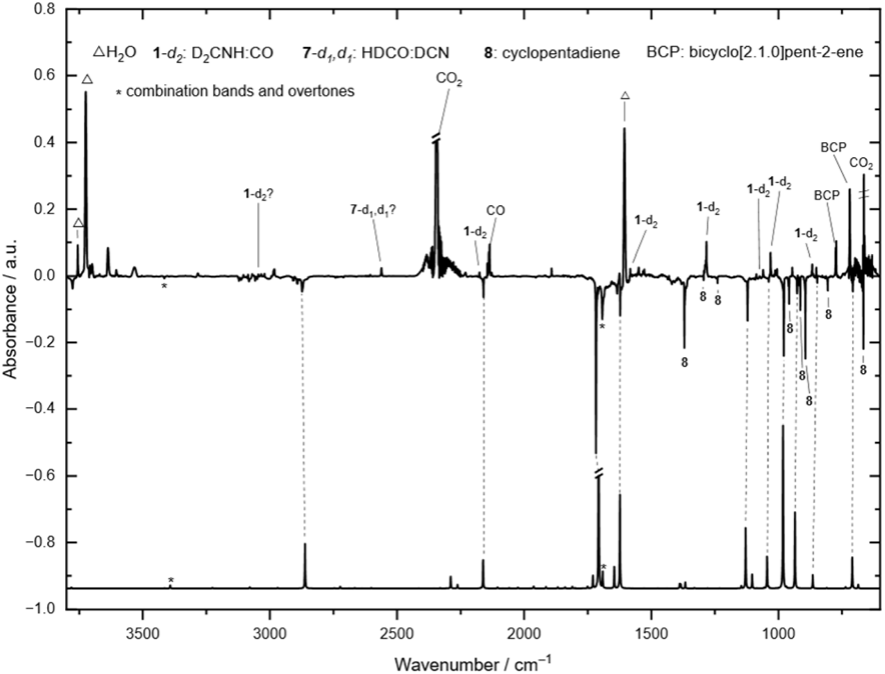
Experimental IR difference spectrum between the matrix IR spectrum at 3.4 K recorded after 32 min pyrolysis of 5 at 450 °C and irradiation of the matrix with UV light (*λ* = 308–254 nm) for 270 min. Bottom: Calculated anharmonic IR spectrum of *gauche*-**4**-*d*_*2*_ at the B2PLYP/6-311++G(2d,2p) level of theory. After UV irradiation *gauche*-**4**-*d*_2_ decomposes into formaldimine-*d*_*2*_:CO (**1**-*d*_*2*_) and DCN:formaldehyde-d_1_ (**7**-*d*_1_,*d*_1_).

To provide further spectroscopic evidence for the generation of *gauche*-4, we synthesized 3,3-deuterium labelled *N*-formyl-2-azabicyclo[2.2.1]hept-5-ene (5-*d*_2_). The compound can easily be prepared by reducing Vince lactam^42^ (2-azabicyclo[2.2.1]hept-5-en-3-one, 9) with lithium aluminum deuteride (LiAlD_4_) and subsequent formylation (Scheme S1†).^20^ We carried out pyrolysis experiments with the deuterium labeled compound in a similar fashion and assigned a new set of signals at 2281.0, 2159.8, 1711.9, 1623.3, 1121.4, 1039.1, 979.3, 926.6, 847.6, and 707.8 cm^−1^ to deuterium labeled *gauche*-4-*d*_2_ (D_2_CNHCO) based on our CCSD(T)/cc-pVTZ frequency calculations (Tables 2 and S2† (extended version), Fig. 2). Irradiation of the matrix with 254 nm results in the disappearance of the described signals and the formation of complexed formaldimine-*d*_2_ (ref. 38) (D_2_CNH) and CO as well as a DCN:formaldehyde-*d*_1_ complex. The tiny signal at 3415.8 cm^−1^, which is marked with an asterisk, is assigned to the overtone signal of the fundamental of *gauche*-4-*d*_2_ at 1717.6 cm^−1^. The characteristic marked signal in the carbonyl region at 1692.9 cm^−1^ is a combinational band of the fundamentals at 707.8 and 979.3 cm^−1^.

**Table 2:** Comparison of experimental fundamental vibrational frequencies of gauche-4-*d*_2_ isolated in argon at 3.4 K and calculated fundamental vibrational frequencies^a^

Mode	Approx. description	B2PLYP/6-311++G(2d,2p)		Experiment	
		*ṽ*_anh._/cm^−1^	*I*_anh._/km mol^−1^	*ṽ*_e*x*p._/cm^−1^	*I*_rel_
4	*τ*(DCNC), *δ*(NCO)	709.7	10.7	707.8	w
5	*τ*(NCDD)	865.0	5.5	847.6	w
6	*δ*(DCD), *δ*(HCO)	934.9	33.7	926.6	w
7	*δ*(NCD), *δ*(DCD)	982.1	76.9	979.3	m
8	*τ*(NCHO)	1044.8	16.3	1039.1	w
9	*δ*(DCD)	1129.0	32.3	1121.4	m
10	*δ*(HCO)	1366.5	4.0	1368.7°	w
11	*v*(CN)	1623.5	71.9	1623.3	m
12	*v*(CO)	1707.2	193.9	1717.6	s
13	*v*_sym_(CD_2_)	2161.7	29.9	2159.8	w
14	*v*_as_(CD_2_)	2289.0	12.9	Ov.°, (CO_2_)	w
15	*v*(CH)	2861.8	63.5	2871.4	w

a rel. experimental intensities (w = weak, m = medium, s = strong); ° = overlap.

### UV/Vis spectroscopy

**Fig. 3:**
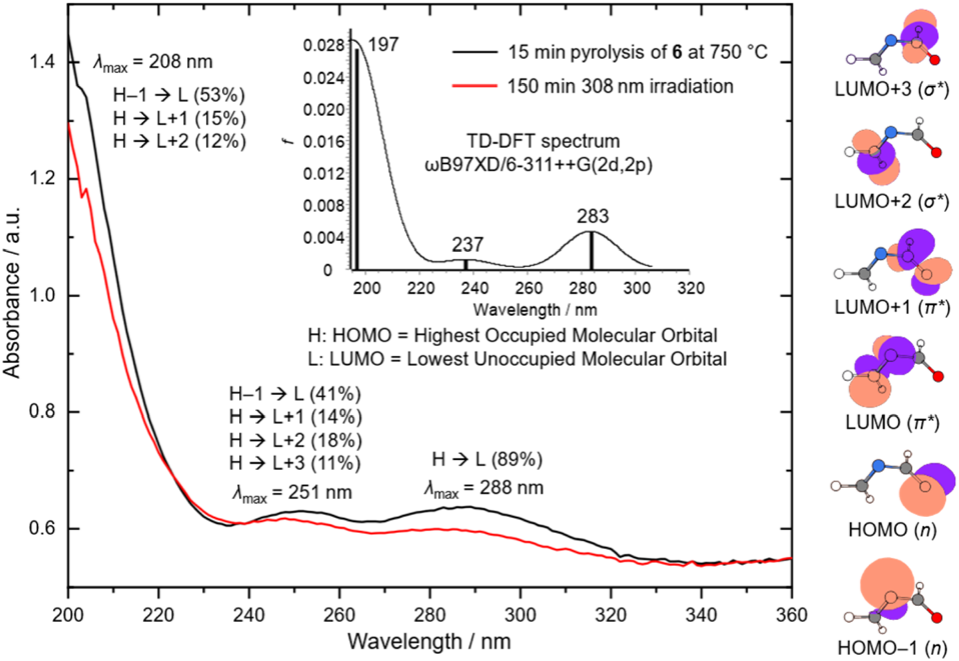
Experimental matrix isolation UV/Vis spectrum at 3.4 K after 15 min pyrolysis of 6 (black curve) at 750 °C and after 150 min UV irradiation (*λ* = 308 nm) (red curve). Inset top: Calculated TD-DFT spectrum of 4 at the TD-ωB97XD/6-311++G(2d,2p) level of theory. Right column: Natural bond orbitals of 4 (contour value: 0.06) calculated at the TD-ωB97XD/6-311++G(2d,2p) level of theory. Color code: carbon: grey; hydrogen: white; nitrogen: blue; oxygen: red (covered by orbitals).

We also repeated the pyrolysis experiments with 6 and conducted UV/Vis spectroscopy experiments with the trapped pyrolysis products. As the UV/Vis absorptions of 8 show critical overlaps in the UV region with the calculated transitions of *gauche*-4, precursor 6 is more suitable for UV/Vis experiments than 5. The major pyrolysis side product, ethylene, shows absorption below 200 nm,^43^ as well as the other minor by-products formaldehyde,^44^ HCN,^45^ and CO.^46^ Only methylene imine has a transition at around 250 nm;^47^ however, in the pyrolysis IR spectrum it is observed only in traces (Fig. S3†). Precursor 6 has a calculated weak transition at *λ* = 206 nm (oscillator strength *f* = 0.007) at the TD-ωB97XD/6-311++G(2d,2p)^34,35,48^ level of theory that is in very good agreement with our experimental UV/Vis spectrum in argon (*λ*_ma*x*_ = 211 nm, Fig. S9†). For *gauche*-4 we calculated transitions at the same level of theory at *λ* = 283 nm (*f* = 0.005), *λ* = 237 nm (*f* = 0.001), and *λ* = 196 nm (*f* = 0.028) (Fig. 3). After 15 min pyrolysis of 6, we recorded the black spectrum depicted in Fig. 3 with three absorption maxima at *λ*_max_ = 288 nm, *λ*_max_ = 251 nm, and *λ*_ma*x*_ = 208 nm. The two absorption bands at *λ*_ma*x*_ = 288 and *λ*_ma*x*_ = 251 nm are in good agreement with the calculated transition while the absorption maximum at *λ*_ma*x*_ = 208 nm overlaps with the absorption of undecomposed precursor 6 (Fig. S9†). After irradiation of the matrix with *λ* = 308 nm for 150 min all bands decreased while no new signals appeared (*vide supra*) as depicted in the red curve in Fig. 3. According to our TD-DFT calculations the electronic transition at 283 nm can be associated with a HOMO (Highest Occupied Molecular Orbital)–LUMO (Lowest Unoccupied Molecular Orbital) transition (89%). The HOMO represents a non-bonding (n) electron pair of oxygen, while the LUMO is the π* orbital of the C=N double bond based on the depicted natural bond orbitals in Fig. 3. Hence, the HOMO–LUMO transition can be attributed to an n–π* transition. The calculated transitions at 237 and 208 nm are more mixed. The major contribution for both transitions comes from the HOMO−1 to LUMO transition (n–π*, 41% and 53%) while there are minor contributions from the HOMO–LUMO+1 (n–π*, 14% and 15%) and the HOMO–LUMO+2 (n–σ*, 18% and 12%) transitions.

### Computational analysis

**Fig. 4:**
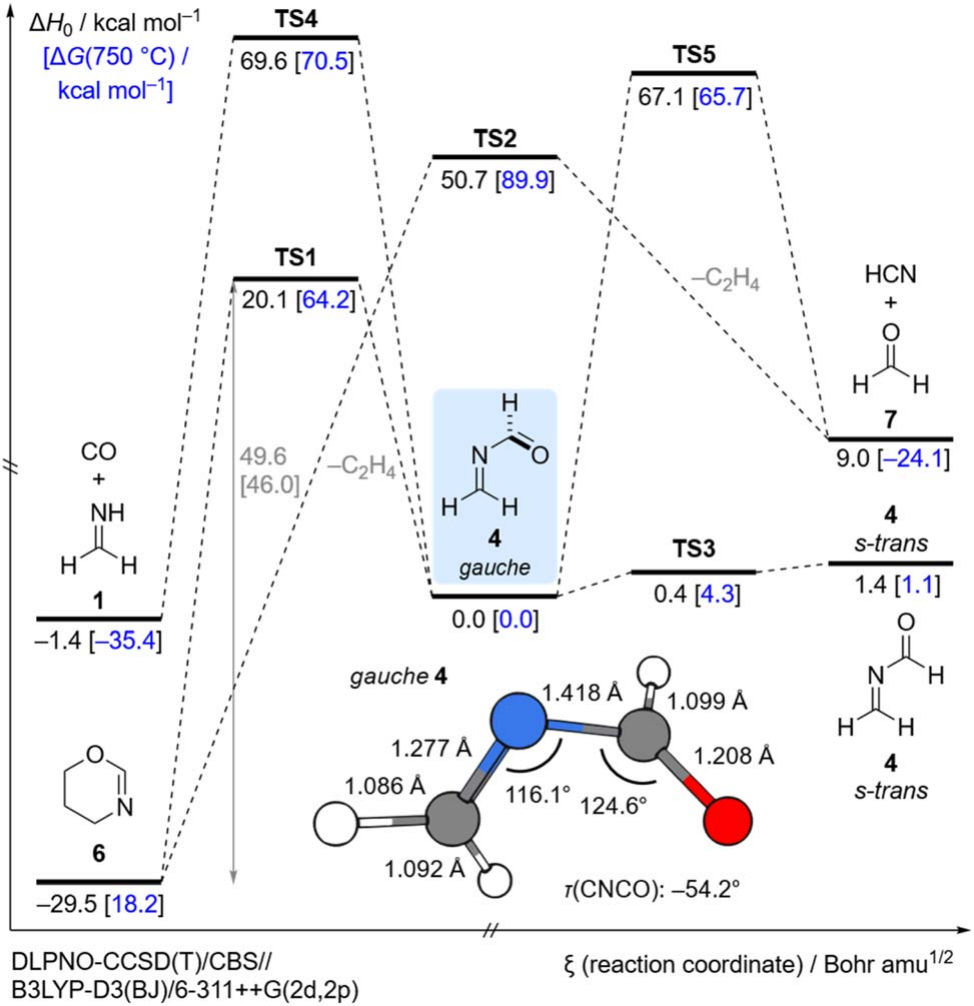
Most essential part of the potential energy surface (PES) around *N*-methyleneformamide (4) at the DLPNO-CCSD(T)/CBS//B3LYP-D3(BJ)/6-311++G(2d,2p) level of theory including zero-point vibrational energy (ZPVE) corrections (Δ*H*_0_) and Gibbs free energy corrections at 750 °C (Δ*G*(750 °C), blue). The depicted geometry of *gauche*-4 is optimized at the CCSD(T)/cc-pVTZ level of theory. Color code: carbon: grey; hydrogen: white; nitrogen: blue; oxygen: red.

We calculated the closed-shell thermal decomposition pathways starting from 5 (Fig. S30†) and 6 (Fig. 4) that lead to the formation of 4 at the DLPNO-CCSD(T)/CBS//B3LYP-D3(BJ)/6-311++G(2d,2p)^49–56^ level of theory including zero-point vibrational energy (ZPVE) corrections (Δ*H*_0_) and Gibbs free energy corrections at 750 °C (Δ*G*(750 °C), blue in Fig. 4). The Gibbs barrier for the decomposition of 6 at 750 °C in the retro-aza-Diels–Alder reaction is 46.0 kcal mol^−1^ (TS1) and is associated with the formation of 4 and ethylene. We located another transition state TS2 that is much higher in energy (89.9 kcal mol^−1^) and connects precursor 6 directly to 7, CO and ethylene. The imaginary frequency of TS2 resembles a ring breathing vibration in 6. However, a stepwise decomposition of 6*via gauche*4 and TS5 (65.7 kcal mol^−1^) is more favorable at the high pyrolysis temperatures. We located another transition state TS4 (70.5 kcal mol^−1^) that is associated with the dissociation of 4 into 1 and CO. All dissociation products were also identified in our pyrolysis spectrum at elevated temperatures. For 5 the Gibbs barrier for the retro-aza-Diels–Alder reaction at 450 °C is 41.0 kcal mol^−1^ (TS6, Fig. S29†). This is in good agreement with our experimental results, as 5 already decomposed at much lower pyrolysis temperatures (*vide supra*).

**Fig. 5:**
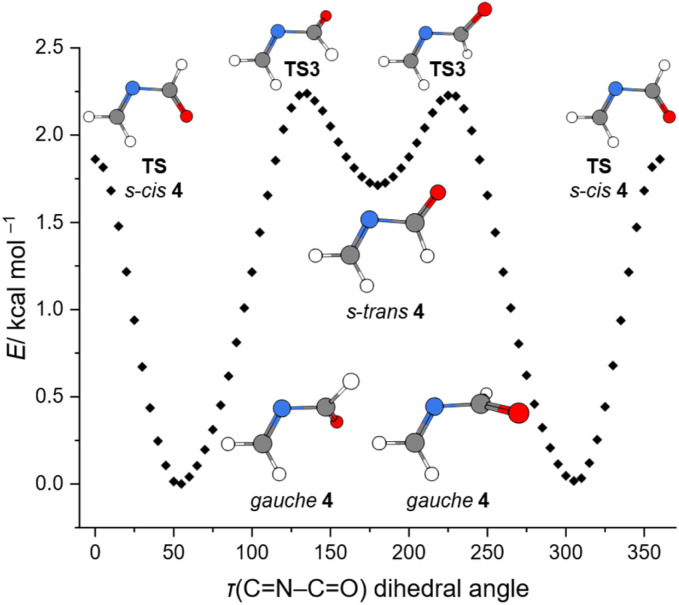
Rotational profile around the C=N–C=O dihedral angle of *N*-methyleneformamide (4) calculated at the DLPNO-CCSD(T)/CBS//B3LYP-D3(BJ)/6-311++G(2d,2p) level of theory.

Our matrix isolation experiments were conducted at 3.4 K. At this low temperature thermal contributions to the Gibbs free energy are negligible and our experiments are well represented by ZPVE corrected electronic energies (Δ*H*_0_). There are two conformers for 4, namely *gauche* and s-*trans*. The planar s-*trans* conformer is 1.4 kcal mol^−1^ higher in energy, while the planar s-*cis* conformation represents a transition state (0.4 kcal mol^−1^), which connects two *gauche* minima of 4 in a double valley. We performed a relaxed scan of the electronic energy of the *τ*(CNCO) dihedral angle in 4 at the DLPNO-CCSD(T)/CBS//B3LYP-D3(BJ)/6-311++G(2d,2p) level of theory. The obtained rotational profile is shown in Fig. 5. The dihedral angle *τ*(CNCO) in *gauche*4 is (−)54.2° calculated at the CCSD(T)/cc-pVTZ level of theory (Fig. 4). However, at the chosen DFT level the *τ*(CNCO) dihedral angle for the optimized geometry deviates by almost 10° and is (−)64.6°, which is a bit larger than with the B2PLYP functional ((−)61.2). The *gauche* and s-*trans* conformers of 4 are connected *via*TS3. With augmented coupled cluster energies and ZPVE corrections we obtain a barrierless transition in Fig. 4. This demonstrates the dependency of the augmented coupled cluster energy on the molecular geometry and ZPVE corrections (Fig. 5). The small barrier and the high energy nature of s-*trans*4 (1.4 kcal mol^−1^) are a reasonable explanation of why we only observed the *gauche* conformer in our low temperature matrix isolation experiments.

## Conclusions

In summary, we report the full matrix isolation IR and UV/Vis spectroscopic characterization of *gauche N*-methyleneformamide and its photochemical UV light induced fragmentation into formaldehyde:HCN and formaldimine:CO complexes. As further decomposition products we observed.

HCN:CO and HNC:CO complexes. Theoretical explorations with multiconfigurational methods on such photochemical decompositions could shed some more light on the detailed reaction mechanisms in the future.

In contrast to the parent 1,3-butadiene *N*-methyleneformamide prefers a *gauche* over an s-*trans* conformation. *N*-Methyleneformamide is a promising candidate for interstellar detection. To facilitate an interstellar search, the molecule should be characterized by rotational spectroscopy to provide the necessary laboratory rotational transitions. If this compound is formed in laboratory astrochemistry experiments or is directly observed in space, it would suggest a possible connection between simple acylimines and larger biologically relevant molecules, such as cytosine as a nucleobase in which the sequence R_2_C=N–RC=O appears. This discovery could potentially increase the complexity of prebiotic molecules of interest to astrobiology and the search for the molecular origins of life.

## Author contributions

V. P. and D. K. synthesized the precursors. V. P. and V. D. D. conducted all matrix isolation experiments. V. P. and A. K. E. performed the computational studies, analyzed the data and co-wrote the manuscript. A. K. E. conceived the idea and supervised the project.

## Conflicts of interest

There are no conflicts to declare.

## Supplementary Material

SC-016-D5SC02777B-s001

SC-016-D5SC02777B-s002

## Data Availability

The data that support the findings of this study are available in the ESI.† These include selected IR and UV/Vis spectra and data, synthetic procedures, NMR spectra, Cartesian Coordinates and energies of calculated structures. All evaluated spectra are available free of charge electronically at https://doi.org/10.17877/RESOLV-2025-M724WNW4.
