# Supplementary material for: Generation and UV-photolysis of N-methyleneformamide
Source: Chem Sci. 2025 May 15;16(24):11103–9. doi: 10.1039/d5sc02777b (PMC12097145; doi:10.1039/d5sc02777b)
Supplement: SC-016-D5SC02777B-s002 [file SC-016-D5SC02777B-s002.pdf]

Supplementary Information (SI) for Chemical Science.
This journal is © The Royal Society of Chemistry 2025

Supporting Information

Generation and UV-photolysis of *N*-methyleneformamide

Viktor Paczelt, Vladimir D. Drabkin, Daniel Kühn and André K. Eckhardt*

Lehrstuhl für Organische Chemie II, Ruhr-Universität Bochum, 44801 Bochum (Germany); Andre.Eckhardt@ruhr-uni-bochum.de

Table of Contents

Experimental Section S2
Infrared and UV/Vis Spectra S3
IR Spectroscopic Data S10
Synthetic Procedures S12
NMR Spectra S15
Potential Energy Surfaces S24
References S26

Experimental Section

General remarks. NMR spectra were recorded on Bruker AVIII-300 and Neo-400 spectrometers at 303 K. Chemical shifts (δ) are given in ppm relative to tetramethylsilane (TMS, δ = 0.00 ppm) as the internal standard or to the respective solvent residual peaks ($CDCl_3$: δ = 7.26 ppm). ATR FT-IR spectra were recorded using a Shimadzu IRAffinity-1S spectrometer at room temperature collecting 16 scans with a standard resolution of 4.0 cm^{-1} in the 4000–500 cm^{-1} region. HRMS analyses were acquired using a GC-MS system consisting of an Agilent 8890 GC/Q-TOF, in which ionization was achieved by EI.

Matrix isolation studies[1]. All matrix isolation infrared studies were performed on a Sumitomo RDK 408D2 closed-cycle refrigerator cold head powered by a Sumitomo F70H compressor unit. The vacuum shroud was outfitted with transparent KBr windows and the sample holder mounted at the base of the cold head was outfitted with a polished CsI window transparent in the IR measurement range of 4000-400 cm^{-1}. The temperature on the sample holder was measured by a Si diode and could be adjusted by two resistive heating cartridges operated by a Lakeshore 336 temperature controller. All measurements were conducted at 3.4 K unless stated otherwise and a Pfeiffer HiCube 80 Eco turbomolecular pump was employed to obtain a high vacuum of approximately 1 x 10^{-5} mbar at room temperature. The *N*-formyl-2-azabicyclo[2.2.1]hept-5-ene (**5**) and 5,6-dihydro-4H-1,3-oxazine (**6**) precursors were synthesized according to the literature[2,3] (see Supporting information for the detailed procedures). The samples were degassed by repeated freeze–pump–thaw cycles and deposited from a glass flask at 0 °C and –65 °C, respectively. During deposition they were co-condensed onto a CsI window at 12 K with a large excess of argon. The flow rate of the noble gas was set to 2.0 sccm with the help of an MKS mass flow controller. Typically, 1.5 mbar min^{-1} Ar was deposited onto the CsI window from a ~2.5 L silylated glass storage bulb over the course of an hour. Irradiation experiments with 308 nm wavelength were conducted with a Radium Xeradex irradiation system with a XeCl* excimer lamp. For irradiation experiments with a wavelength of 254 nm we employed an Oase Vitronic 11 pool lamp (14 Watt). IR spectra were recorded using a Bruker Invenio X spectrometer collecting 64 scans with a standard resolution of 0.5 cm^{-1} in the 4000-400 cm^{-1} region with the DTGS detector. All recorded IR spectra were processed and visualized with Bruker's OPUS 7.5 software package. For all experiments we used Ar of 99.999% purity.

Quantum chemical calculations. For all geometry optimizations and frequency calculations we employed the Gaussian16 program package.[4] The hybrid functional B3LYP[5–7] in combination with the 6-311++G(2d,2p)[8,9] basis set and Grimme's D3 dispersion correction with Becke–Johnson damping[10] was used for all geometry optimizations of all stationary points depicted on the potential energy surfaces. Vibrational frequency calculations were employed to characterize stationary points (zero imaginary frequencies for minima and exactly one imaginary frequency for transition states), determine zero-point vibrational energy corrections, and compute harmonic infrared spectra for comparison with experimental data. All structures were visualized using Chemcraft.[11] Coupled cluster complete basis set extrapolations were performed with orca 5.0.4.[12] Coupled cluster geometry optimization and frequency calculations were conducted with cfour 2.1.[13] Anharmonic frequencies were calculated with Grimme's B2PLYP semiempirical hybrid density functional with perturbative second-order correlation.[14] TD-DFT spectra were calculated with the ωB97XD functional.[15]

Infrared and UV/Vis Spectra

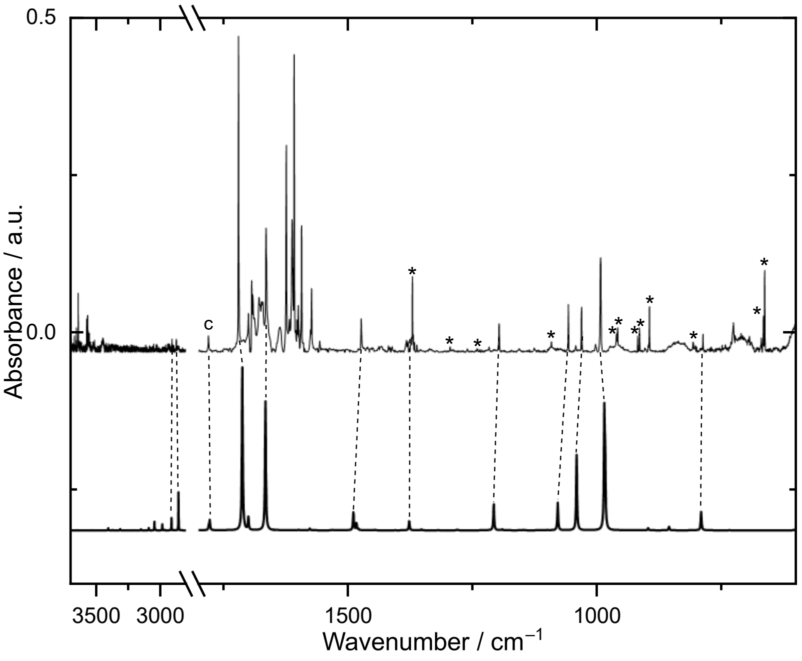

Figure S1: Top: IR spectrum after the pyrolysis of **5** at 450°C isolated in Ar matrix at 3.4 K. Bottom: Calculated IR spectrum of *gauche*-*N*-methyleneformamide (**4**) at the B2PLYP/6-311++G(2d,2p) level of theory. Labels: (*) Cyclopentadiene (**8**), (combi) Combination band of *gauche*-*N*-methyleneformamide (**4**).

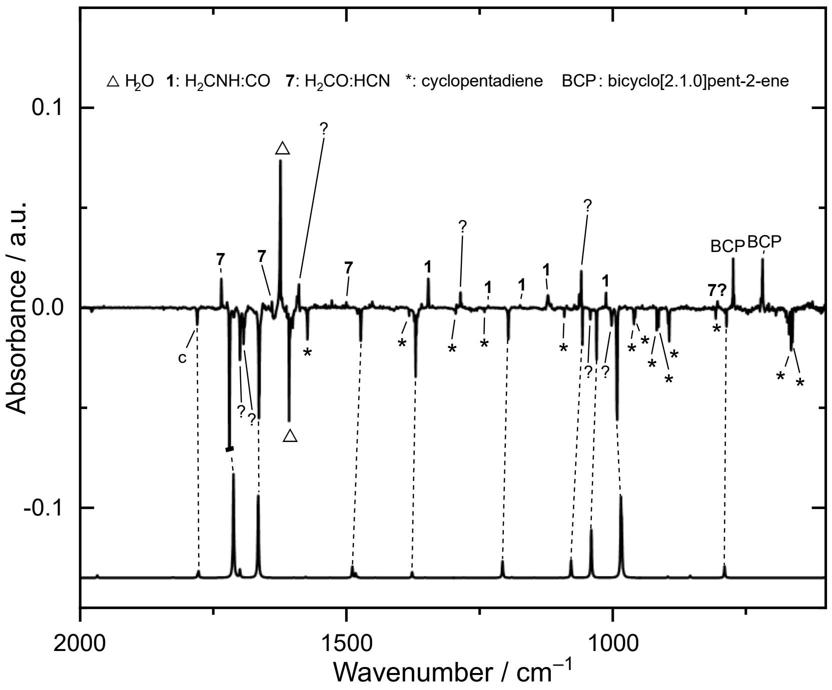

Figure S2a: Difference spectrum of the spectrum depicted in Figure S1 and the spectrum recorded after 40 min 254 nm irradiation. Bottom: Calculated IR spectrum of *gauche*-*N*-methyleneformamide (**4**) at the B2PLYP/6-311++G(2d,2p) level of theory. Labels: (combi) combination band of **4**.

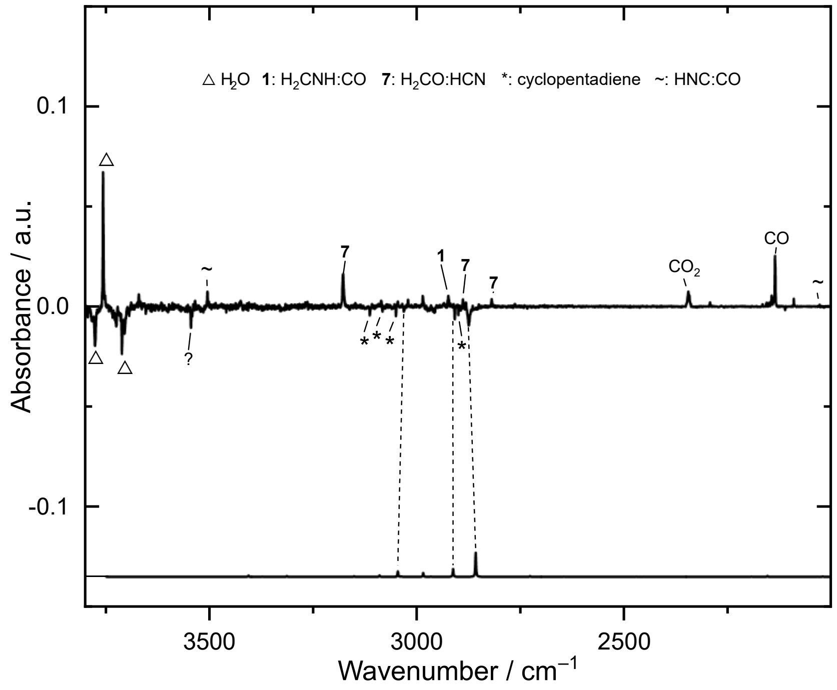

Figure S2b: Difference spectrum of the spectrum depicted in Figure S1 and the spectrum recorded after 40 min 254 nm irradiation. Bottom: Calculated IR spectrum of *gauche*-*N*-methyleneformamide (**4**) at the B2PLYP/6-311++G(2d,2p) level of theory. Labels: (combi) combination band of **4**.

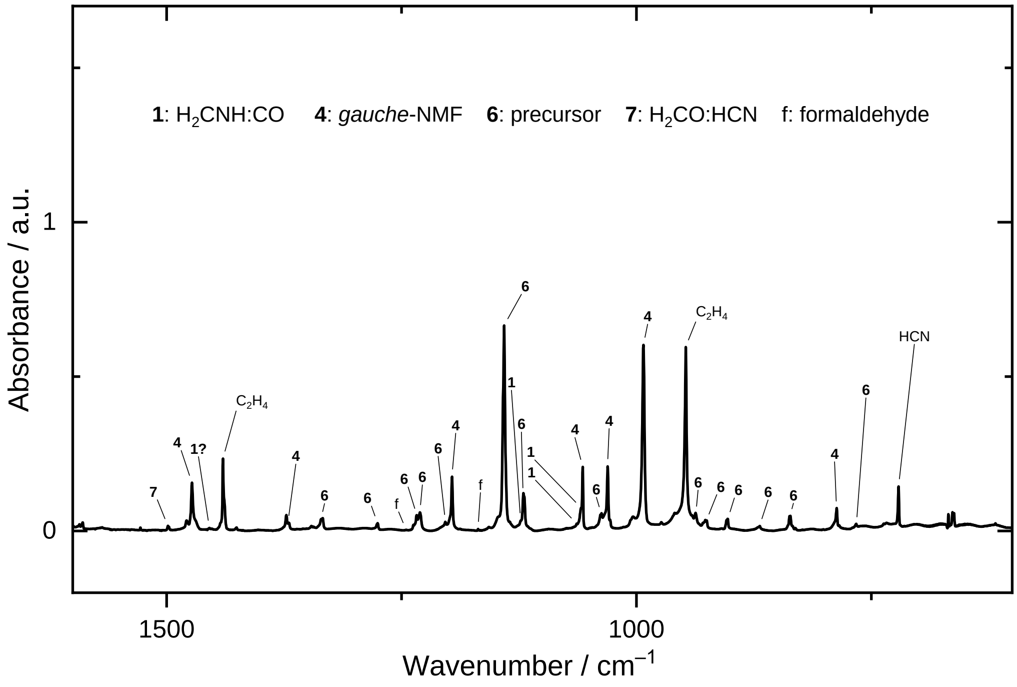

Figure S3a: IR spectrum after the pyrolysis of 5,6-dihydro-4H-1,3-oxazine (**6**) at 750°C isolated in solid Ar at 3.4 K.

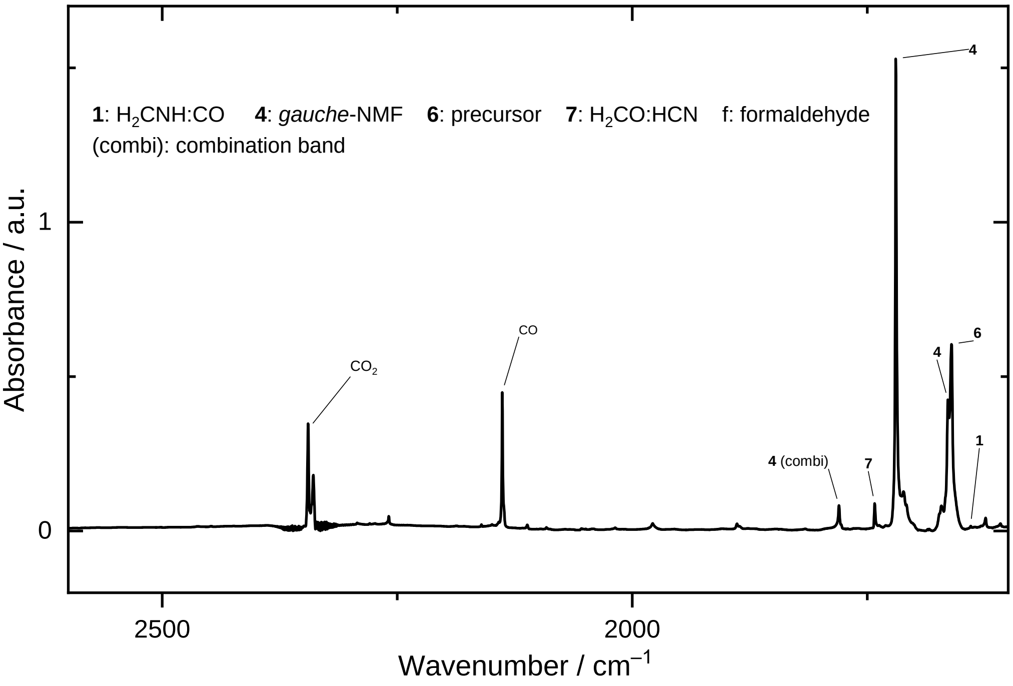

Figure S3b: IR spectrum after the pyrolysis of 5,6-dihydro-4H-1,3-oxazine (**6**) at 750°C isolated in Ar matrix. The observed combination band at 1780 cm^{-1} is the result of simultaneous excitation of fundamental vibrations of the mode 4 and 5 (Table S1).

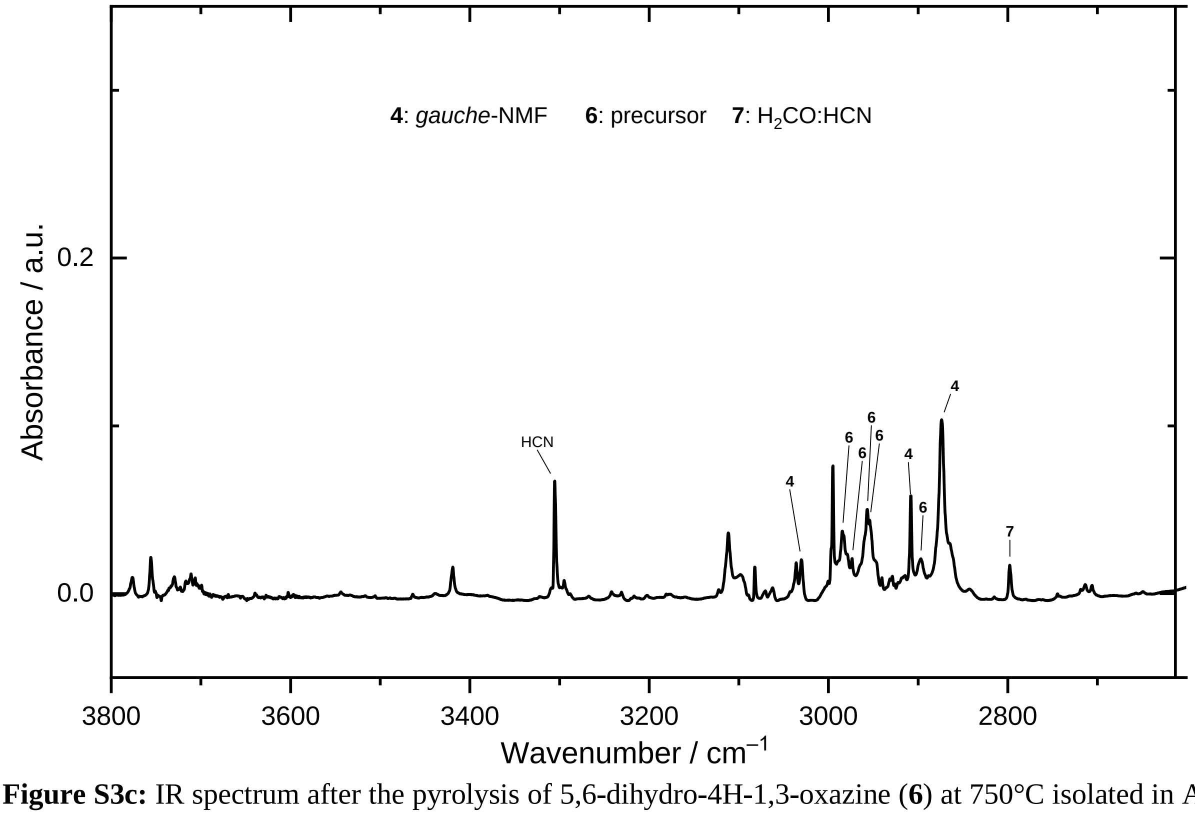

Figure S3c: IR spectrum after the pyrolysis of 5,6-dihydro-4H-1,3-oxazine (**6**) at 750°C isolated in Ar matrix.

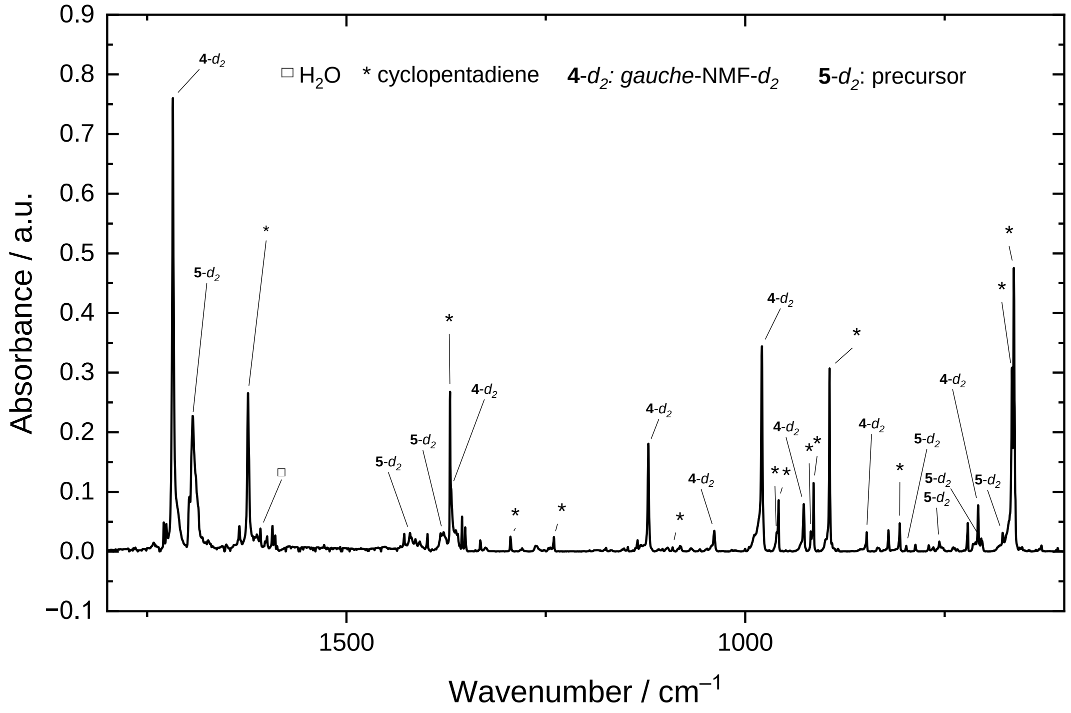

Figure S4a: IR spectrum after the pyrolysis of **5-**d_2 at 450°C isolated in Ar matrix at 3.4 K.

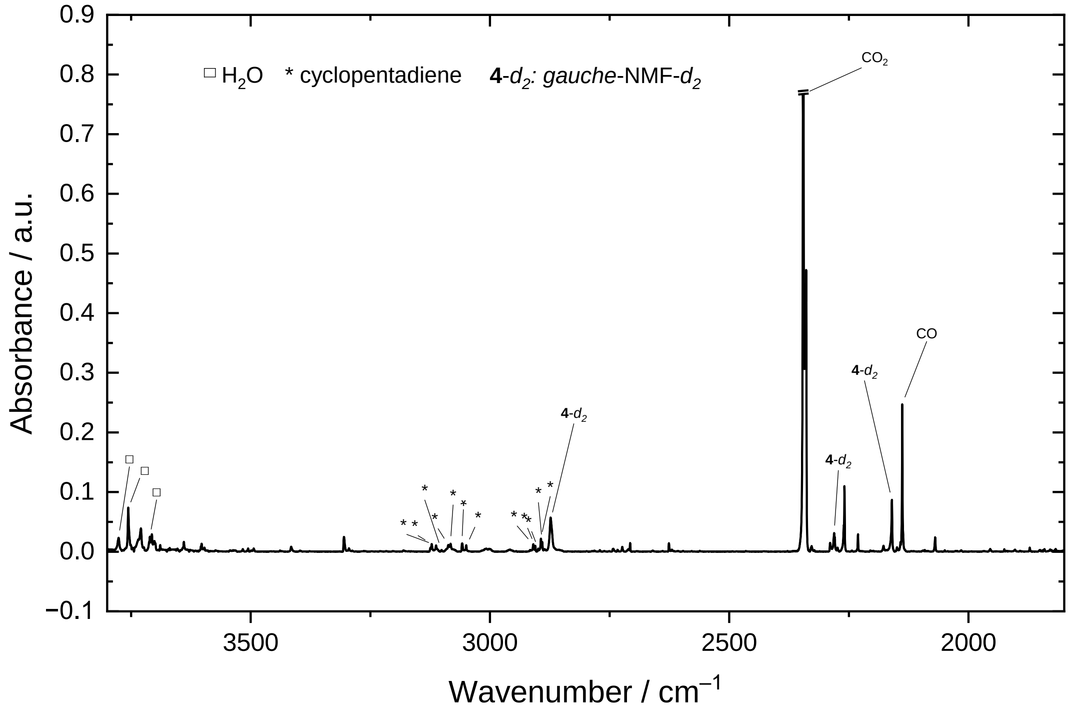

Figure S4b: IR spectrum after the pyrolysis of **5-**d_2 at 450°C isolated in Ar matrix at 3.4 K.

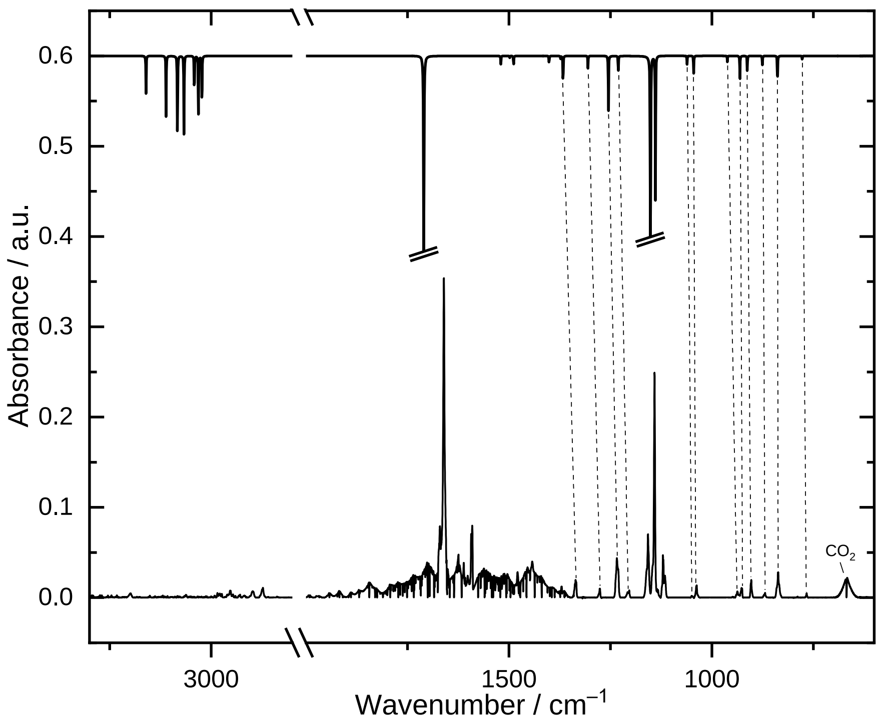

Figure S5: Top: Calculated harmonic IR spectrum of 5,6-dihydro-4H-1,3-oxazine (**6**) at the B3LYP/ 6-311++G(2d,2p) level of theory. Bottom: IR spectrum of the deposited **6** in solid argon at 3.4 K.

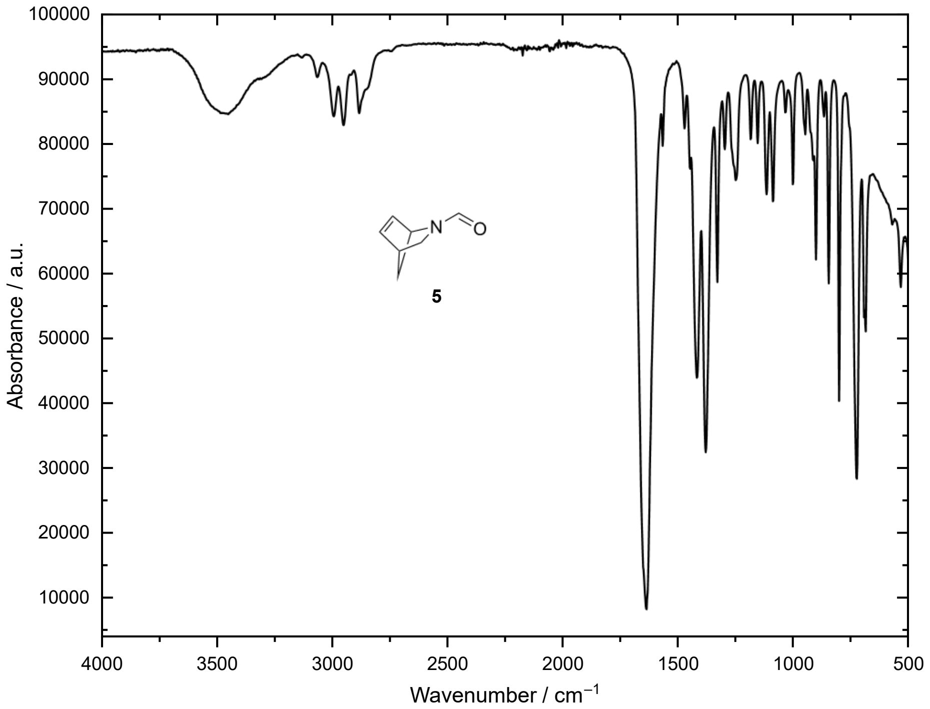

Figure S6: ATR-IR spectrum of *N*-formyl-2-azabicyclo[2.2.1]hept-5-ene (**5**).

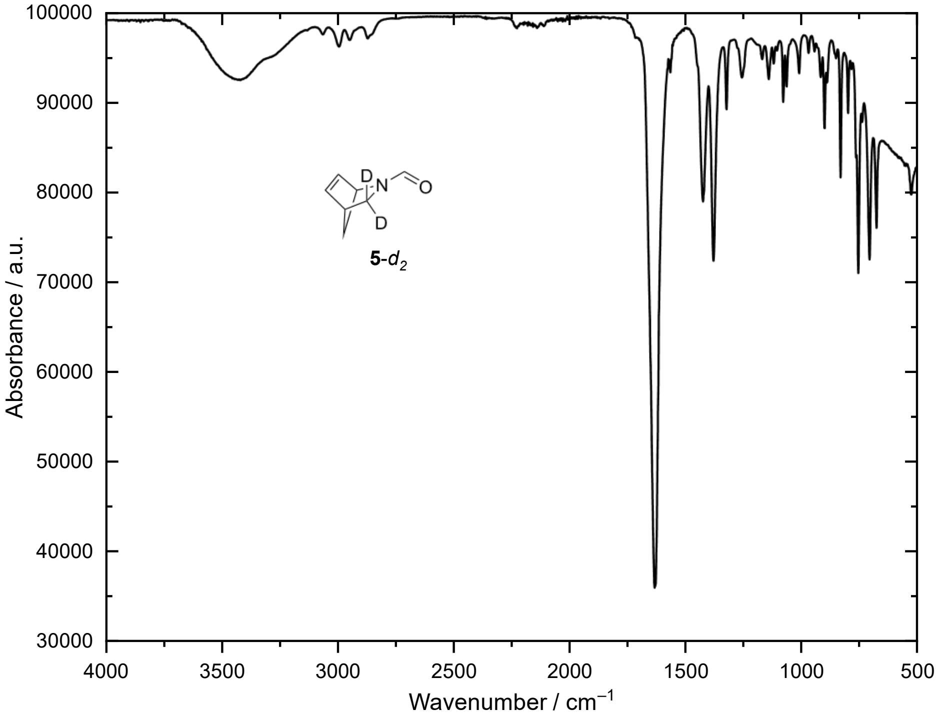

Figure S7: ATR-IR spectrum of *N*-formyl-2-azabicyclo[2.2.1]hept-5-ene-d_2 (**5**-d_2).

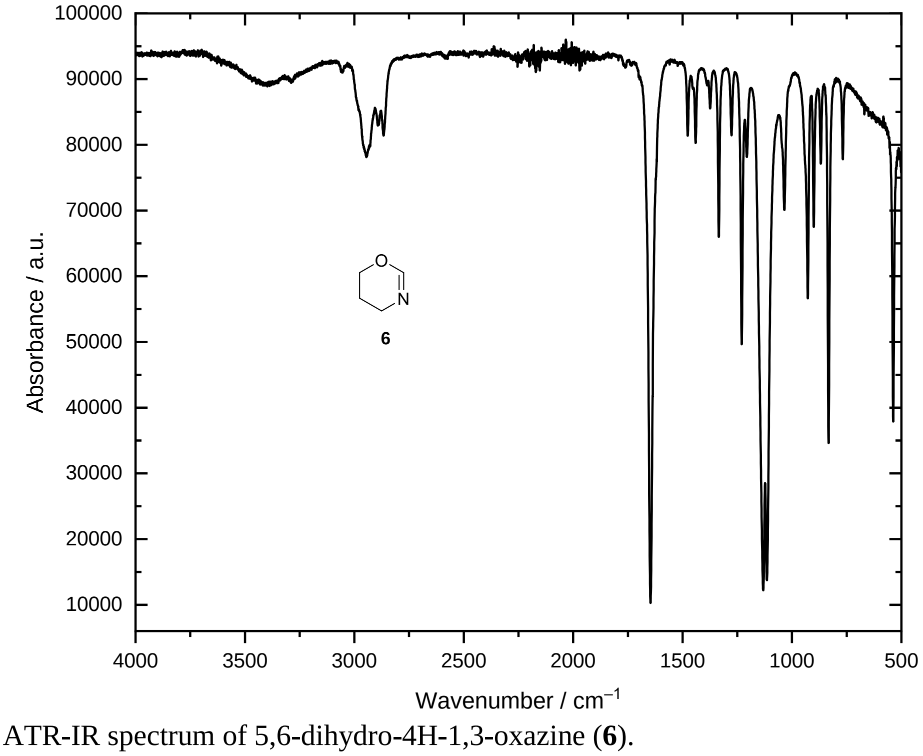

Figure S8: ATR-IR spectrum of 5,6-dihydro-4H-1,3-oxazine (**6**).

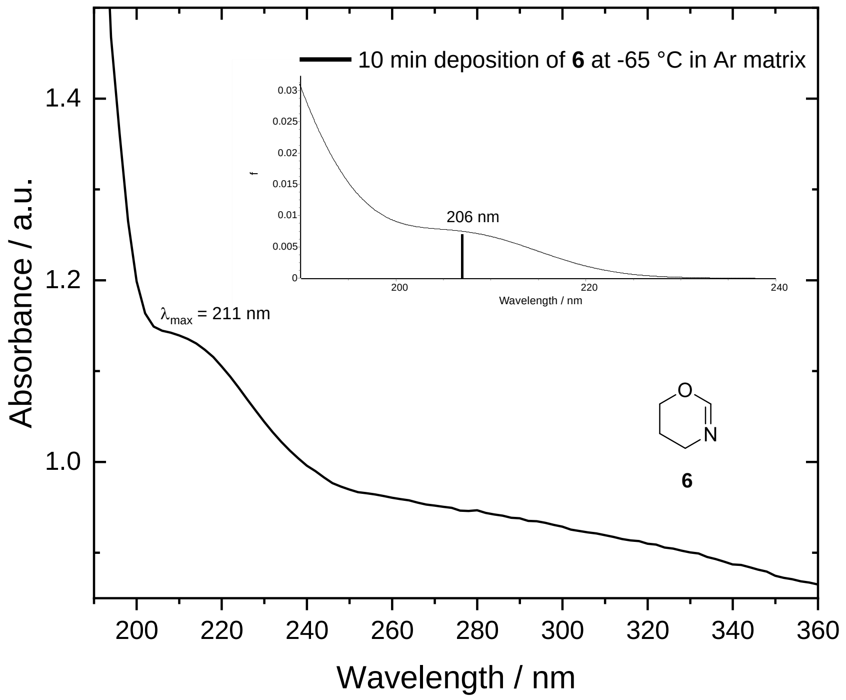

Figure S9: Experimental matrix isolation UV/Vis spectrum at 3.4 K after 10 min deposition of **6** at –65 °C. Inset: Calculated TD-DFT spectrum of **6** at the TD-wB97XD/6-311++G(2d,2p) level of theory.

IR Spectroscopic Data

Table S1: Comparison of experimental fundamental vibrational frequencies of *gauche*-**4** isolated in Ar at 3.4 K and calculated fundamental vibrational frequencies.

Mode	Approx. description	CCSD(T))/ cc-pVTZ		B2PLYP/6-311++G(2d,2p)				Experiment	
		$\tilde{\nu}_{har.}$ / cm^{-1}	$I_{har.}$ / km mol^{-1}	$\tilde{\nu}_{har.}$ / cm^{-1}	$I_{har.}$ / km mol^{-1}	$\tilde{\nu}_{anh.}$ / cm^{-1}	$I_{anh.}$ / km mol^{-1}	$\tilde{\nu}_{exp.}$ / cm^{-1}	$I_{rel.}$
3	δ(NCO), τ(HCNC)	524.8	8.1	506.6	10.7	502.9	7.0	n.o.	-
4	τ(HCNC)	805.1	8.0	808.7	8.2	790.5	8.6	786.6	w
5	ν(CN), δ(NCO), τ(NCHO)	1010.4	101.5	1010.3	113.9	985.0	64.0	992.6	s
6	τ(NCHO)	1057.2	25.5	1059.8	25.8	1040.6	46.1	1030.5	m
7	τ(NCHH)	1085.9	17.1	1099.5	17.6	1078.5	17.3	1057.0	m
8	δ(HCN)	1225.1	16.9	1229.8	20.3	1207.1	18.6	1196.1	m
9	δ(HCO)	1405.3	11.9	1407.2	8.8	1376.8	7.7	1372.6	w
10	δ(HCH)	1500.1	26.0	1518.1	23.7	1488.9	15.6	1473.1	m
11	ν(CN)	1685.4	74.2	1704.9	135.4	1665.6	127.8	1664.0	s
12	ν(CO)	1769.8	173.4	1743.9	194.6	1712.1	163.3	1719.4	vs
13	ν(CH)	3023.2	69.2	3017.3	64.5	2858.0	63.0	2874.7	m
14	$\nu_{sym}(CH_2)$	3066.3	18.7	3073.6	26.1	2912.4	21.5	2908.0	w
15	$\nu_{as}(CH_2)$	3184.2	13.4	3190.9	12.7	3045.6	15.5	3029.9	w

rel. experimental intensities (vw = very weak, w = weak, m = middle, s = strong, vs = very strong); n.o.: not observed

Table S2: Comparison of experimental fundamental vibrational frequencies of gauche-**4**-d_2 isolated in argon at 3.4 K and calculated fundamental vibrational frequencies.

**Mode**	**Approx. description**	**CCSD(T))/ cc-pVTZ**		**B2PLYP/6-311++G(2d,2p)**				**Experiment**	
		$\tilde{\nu}_{har.}$ / cm^{-1}	$I_{har.}$ / km mol^{-1}	$\tilde{\nu}_{har.}$ / cm^{-1}	$I_{har.}$ / km mol^{-1}	$\tilde{\nu}_{anh.}$ / cm^{-1}	$I_{anh.}$ / km mol^{-1}	$\tilde{\nu}_{exp.}$ / cm^{-1}	$I_{rel.}$
4	τ(DCNC), δ(NCO)	716.0	11.5	721.4	12.0	709.7	10.7	707.8	w
5	τ(NCDD)	868.3	5.5	879.4	5.5	865.0	5.5	847.6	w
6	δ(DCD), δ(HCO)	948.4	30.0	951.5	29.9	934.9	33.7	926.6	w
7	δ(NCD), δ(DCD)	1007.8	67.9	1005.6	85.4	982.1	76.9	979.3	m
8	τ(NCHO)	1066.8	10.7	1064.3	10.1	1044.8	16.3	1039.1	w
9	δ(DCD)	1138.2	45.8	1149.1	44.8	1129.0	32.3	1121.4	m
10	δ(HCO)	1404.3	12.3	1406.2	9.2	1366.5	4.0	1368.7 ov.	w
11	ν(CN)	1637.5	69.9	1662.4	92.1	1623.5	71.9	1623.3	m
12	ν(CO)	1766.9	185.9	1738.5	243.1	1707.2	193.9	1717.6	s
13	$\nu_{sym}(CD_2)$	2238.2	23.5	2242.6	30.2	2161.7	29.9	2159.8	w
14	$\nu_{as}(CD_2)$	2374.5	12.2	2379.2	12.1	2289.0	12.9	ov.	w
15	ν(CH)	3023.5	64.8	3017.3	61.0	2861.8	63.5	2871.4	w

rel. experimental intensities (vw = very weak, w = weak, m = middle, s = strong, vs = very strong); n.o.: not observed; ov. = overlap

Synthetic Procedures

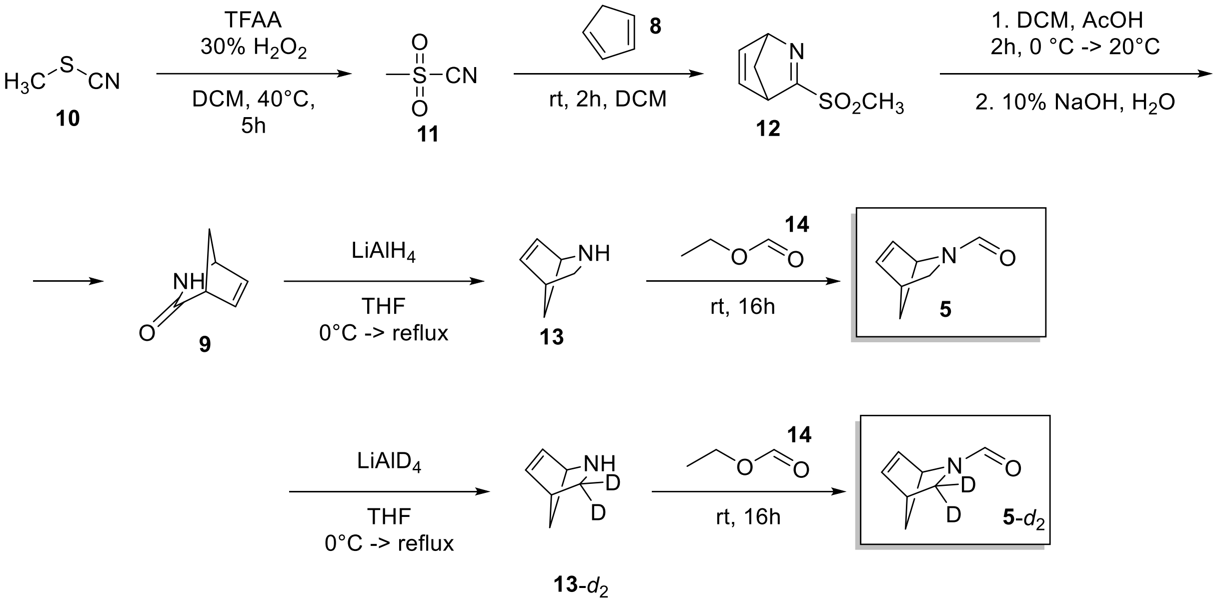

Scheme S1: Synthesis of precursor **5** and isotopically labeled precursor **5**-d_2.

Synthesis of methanesulfonylcyanide (11)
The compound was synthesized according to the literature.[16] To a solution of trifluoroacetic anhydride (86.1g, 410 mmol) in DCM (120 mL) cooled to °0 C was added hydrogen peroxide (46 mL, 410 mmol, aq 30% w/w) dropwise. After the addition, the mixture was stirred for 40 minutes at 0 °C. Methylthiocyanate (**10**) (3.0 g, 41 mmol) was then added dropwise and the mixture was heated to 40 °C for 5 hours. The reaction was then cooled to 0 °C and quenched with ice water (250 mL) and was let warm to room temperature. The organic layer was separated and aqueous one extracted twice with DCM (2x100 mL). The combined DCM extracts were combined washed twice with brine and dried over $MgSO_4$. The solvent was removed at reduced pressure to obtain colorless oil that was stored at -20 °C and used without further purification. Yield: 3.2 g (75%).
^{1}H NMR (300 MHz, $CDCl_3$) δ 3.43 (s, 3H) ppm.
^{13}C{^{1}H} NMR (75 MHz, $CDCl_3$) δ 113.3, 46.7 ppm.

Synthesis of 2-azabicyclo[2.2.1]hept-5-en-3-one (Vince lactam, 9)
The compound was synthesized according to the literature.[17] Freshly distilled cyclopentadiene (**8**) (3.1 g, 46.9 mmol) in DCM (8 mL) cooled to -20 °C was added over 10 min to a solution of sulfonyl cyanide (**11**) (4.1 g, 39 mmol) in DCM (10 mL) at 0 °C. Resulting light yellow solution was stirred at rt for 2 h to obtain the unstable intermediate **12**, which was hydrolysed to **9** as follows: Acetic acid (7 ml, 117 mmol) was then added over 45 min and the solution was stirred for 1 h at rt. The solution was then added dropwise to water (30 mL) in 250 mL beaker kept in room temperature water bath. During the addition pH 8 was kept at around 8 by simultaneous addition of 30% aq. NaOH (ca.16 mL). Phases were then separated and organic phase were extracted with DCM (3 x 20 mL), dried with $MgSO_4$ and solvent removed to obtain brown oily solid that was purified by recrystallization from *n*-Bu_2O or flash column chromatography (DCM/MeOH, 50:1). Yield: 3.1 g (73%) over two steps.
^{1}H NMR (300 MHz, $CDCl_3$) δ 6.83 – 6.75 (m, 1H), 6.72 – 6.63 (m, 1H), 5.54 (s, 1H), 4.38 – 4.29 (m, 1H), 3.26 – 3.16 (m, 1H), 2.44 – 2.36 (m, 1H), 2.27 – 2.18 (m, 1H) ppm.
^{13}C{^{1}H} NMR (75 MHz, $CDCl_3$) δ 185.5, 141.2, 138.2, 60.4, 59.4, 53.3 ppm.

Synthesis of 2-azabicyclo[2.2.1]hept-5-ene (13)

The compound was synthesized according to the literature.[18] The reaction was carried out in oven-dried glassware under argon atmosphere. To a solution of 2-azabicyclo[2.2.1]hept-5-en-3-one (**9**) (1.0 g, 9.2 mmol) in dry THF (15 mL) cooled to 0 °C was slowly added $LiAlH_4$ (0.87 g, 23 mmol). The solution was stirred at room temperature for 30 min and then refluxed for additional 4 hours. After cooling down to room temperature it was diluted with THF (30 mL), quenched carefully with water (1 mL) and lastly 30% NaOH (1.5 mL) was added dropwise. The aluminium salts were filtered off and the cake washed several times with THF. The filtrate was dried with $MgSO_4$ and solvent removed at reduced pressure. The residue was purified by distillation (35°C, 15 mbar) to obtain colorless oil that was stored at -20 °C. Yield: 0.7 g (80%).

^{1}H NMR (300 MHz, $CDCl_3$) δ 6.17 – 6.05 (m, 2H), 3.98 – 3.92 (m, 1H), 3.14 – 3.06 (m, 1H), 3.00 – 2.92 (m, 1H), 2.00 (dd, J = 8.9, 1.5 Hz, 1H), 1.40 – 1.28 (m, 2H) ppm.

^{13}C{^{1}H} NMR (75 MHz, $CDCl_3$) δ 134.2, 134.1, 60.0, 47.7, 44.0, 43.6 ppm.

Synthesis of 2-Azabicyclo[2.2.1]hept-5-ene-2-carboxaldehyde (5)

The compound was synthesized according to the literature.[3] The amine 2-azabicyclo[2.2.1]hept-5-ene (**13**) (0.20 g, 2.10 mmol) was dissolved in 2 mL of ethyl formate. After the solution was stirred overnight, ethyl formate was removed under reduced pressure. The residue was purified by flash column chromatography (DCM/MeOH, 50:1) to obtain colorless oil. Yield: 0.18 g (70%).

Major conformer: ^{1}H NMR (400 MHz, $CDCl_3$) δ 8.29 (s, 1H), 6.40 – 6.24 (m, 2H), 4.58 – 4.53 (m, 1H), 3.44 – 3.36 (m, 1H), 3.32 – 3.25 (m, 1H), 2.80 – 2.75 (m, 1H), 1.74 – 1.60 (m, 2H) ppm.

Minor conformer: ^{1}H NMR (400 MHz, $CDCl_3$) δ 8.14 (s, 1H), 6.40 – 6.24 (m, 2H), 5.07 (s, 1H), 3.56 – 3.50 (m, 1H), 3.30 – 3.26 (m, 1H), 2.85 – 2.81 (m, 1H), 1.68 – 1.54 (m, 2H) ppm.

Major conformer: ^{13}C{^{1}H} NMR (75 MHz, $CDCl_3$) δ 159.8, 137.7, 133.6, 61.5, 48.7, 44.0, 42.5 ppm.

Minor conformer: ^{13}C{^{1}H} NMR (75 MHz, $CDCl_3$) δ 160.8, 137.0, 134.9, 57.7, 47.4, 45.3, 42.4 ppm.

ATR-IR: (1640, 1418, 1380, 1329, 901, 845, 801, 724) cm^{-1}.

Synthesis of 2-azabicyclo[2.2.1]hept-5-ene-d$_2$ (13-*d$_2$*)

The reaction was carried out in oven-dried glassware under argon atmosphere. To a solution of 2-azabicyclo[2.2.1]hept-5-en-3-one (**9**) (350 mg, 3.21 mmol) in dry THF (5 mL) cooled to 0 °C was slowly added $LiADH_4$ (254 mg, 6.7 mmol). The solution was stirred at room temperature for 30 min and then refluxed for additional 4 hours. After cooling down to room temperature it was diluted with THF (30 mL), quenched carefully with water (1 mL) and lastly 30% NaOH (1.5 mL) was added dropwise. The aluminium salts were filtered off and the cake washed several times with THF. The filtrate was dried with $MgSO_4$ and solvent removed at reduced pressure. The crude was used for the next without further purification. Yield: 256 mg (82%).

^{1}H NMR (400 MHz, $CDCl_3$) δ 6.17 – 6.07 (m, 2H), 3.97 – 3.93 (m, 1H), 3.13 – 3.08 (m, 1H), 1.41 – 1.32 (m, 2H) ppm.

^{2}H NMR (61 MHz, $CHCl_3$) δ 2.87 (s, 1H), 1.91 (s, 1H) ppm.

^{13}C{^{1}H} NMR (75 MHz, $CDCl_3$) δ 134.7, 134.6, 60.4, 48.1, 43.9 ppm.

Synthesis of 2-Azabicyclo[2.2.1]hept-5-ene-2-carboxaldehyde-d_2 (5-d_2)

The amine 2-azabicyclo[2.2.1]hept-5-ene-d_2 (**13-d_2**) (200 mg, 2.06 mmol) was dissolved in 2 mL of ethyl formate. After the solution was stirred overnight, ethyl formate was removed under reduced pressure. The residue was purified by flash column chromatography (DCM/MeOH, 50:1) to obtain colorless oil. Yield: 191 mg (74%).

Major conformer: ^{1}H NMR (400 MHz, $CDCl_3$) δ 8.29 (s, 1H), 6.41 – 6.24 (m, 2H), 4.56 (s, 1H), 3.27 (m, 1H), 1.74 – 1.60 (m, 2H) ppm.

Minor conformer: ^{1}H NMR (400 MHz, $CDCl_3$) δ 8.13 (s, 1H), 6.40 – 6.30 (m, 2H), 5.09 – 5.04 (m, 1H), 3.28 – 3.25 (m, 1H), 1.68 – 1.57 (m, 2H) ppm.

Major conformer: ^{2}H NMR (61 MHz, $CHCl_3$) δ 3.38 (1H), 2.75 (1H) ppm.

Minor conformer: ^{2}H NMR (61 MHz, $CHCl_3$) δ 3.51 (1H), 2.80 (1H) ppm.

Major conformer: ^{13}C{^{1}H} NMR (101 MHz, $CDCl_3$) δ 160.0, 137.9, 133.7, 61.6, 48.8, 42.4 ppm.

Minor conformer: ^{13}C{^{1}H} NMR (101 MHz, $CDCl_3$) δ 160.9, 137.1, 135.2, 57.9, 47.6, 42.3 ppm.

ATR-IR: (1633, 1425, 1381, 1323, 901, 832, 755, 707, 672) cm^{-1}.

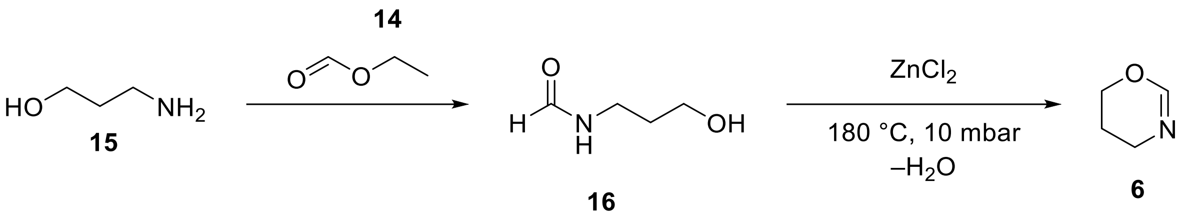

Scheme S2: Synthesis of 5,6-dihydro-4H-1,3-oxazine (**6**).

Synthesis of N-(3-hydroxypropyl)formamide (16)

3-Amino-1-propanol (**15**) (5.0 g, 66.6 mmol) was slowly added to neat ethyl formate (**14**) (10 mL) at 0 °C. The reaction mixture was let to reach room temperature and was stirred overnight. After the removal of the formate, the distillation (220 °C, 5 mbar) with Vigreux column afforded colorless viscous liquid. Yield: 6.0 g (88%).

^{1}H NMR (300 MHz, $CDCl_3$) δ 8.20 (s, 1H), 6.11 (s, 2H), 3.73 – 3.63 (m, 2H), 3.54 – 3.42 (m, 1H), 2.95 – 2.84 (m, 1H), 1.78 – 1.69 (m, 2H) ppm.

^{13}C{^{1}H} NMR (101 MHz, $CDCl_3$) δ 162.3, 59.8, 35.5, 32.0 ppm.

5,6-Dihydro-4H-1,3-Oxazine (6)

The synthetic procedure of the compound was adapted from the literature.[2] Flask containing N-(3-hydroxypropyl)formamide (**16**) (4.0 g, 38.8 mmol) and $ZnCl_2$ (0.6 g, 4.5 mmol) was attached to a short-path distillation apparatus and the system was evacuated to 10 mbar. The stirred reaction mixture was heated to 170 °C for 1 hour during which the mixture of product and water were collected in a receiver cooled to -30 °C. Three rounds of distillation from NaOH pellets at room temperature and 10 mbar and finally one distillation from anhydrous BaOH afforded the product with sufficient purity for matrix isolation. The colorless liquid was stored at -20 °C. Yield: 2.4 g (74%).

^{1}H NMR (300 MHz, $CDCl_3$) δ 7.00 (s, 1H), 4.20 – 4.11 (m, 2H), 3.39 – 3.29 (m, 1H), 2.01 – 1.87 (m, 1H) ppm.

^{13}C{^{1}H} NMR (101 MHz, $CDCl_3$) δ 150.0, 64.4, 41.4, 22.4 ppm.

HRMS (EI-TOF): $[M]^+$ calcd for $C_4H_7NO^+$: 85.0522; found: 85.0524.

NMR Spectra

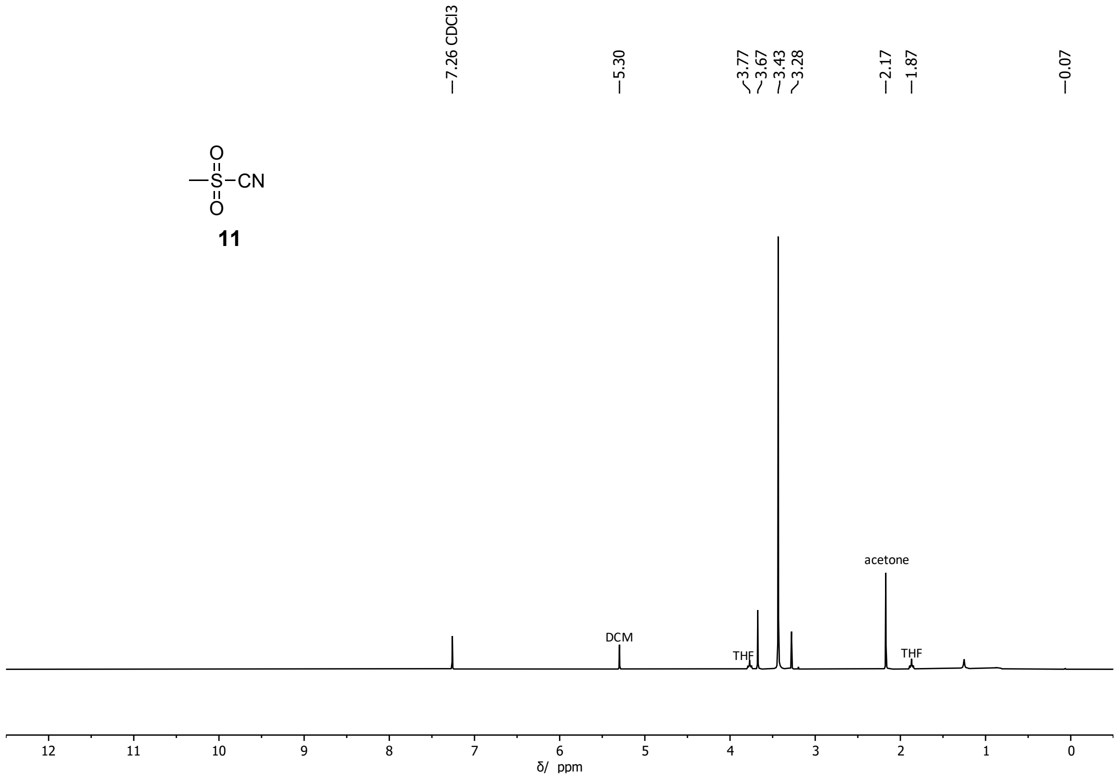

Figure S10: ^{1}H NMR (300 MHz, $CDCl_3$) spectrum of methanesulfonyl cyanide (**11**).

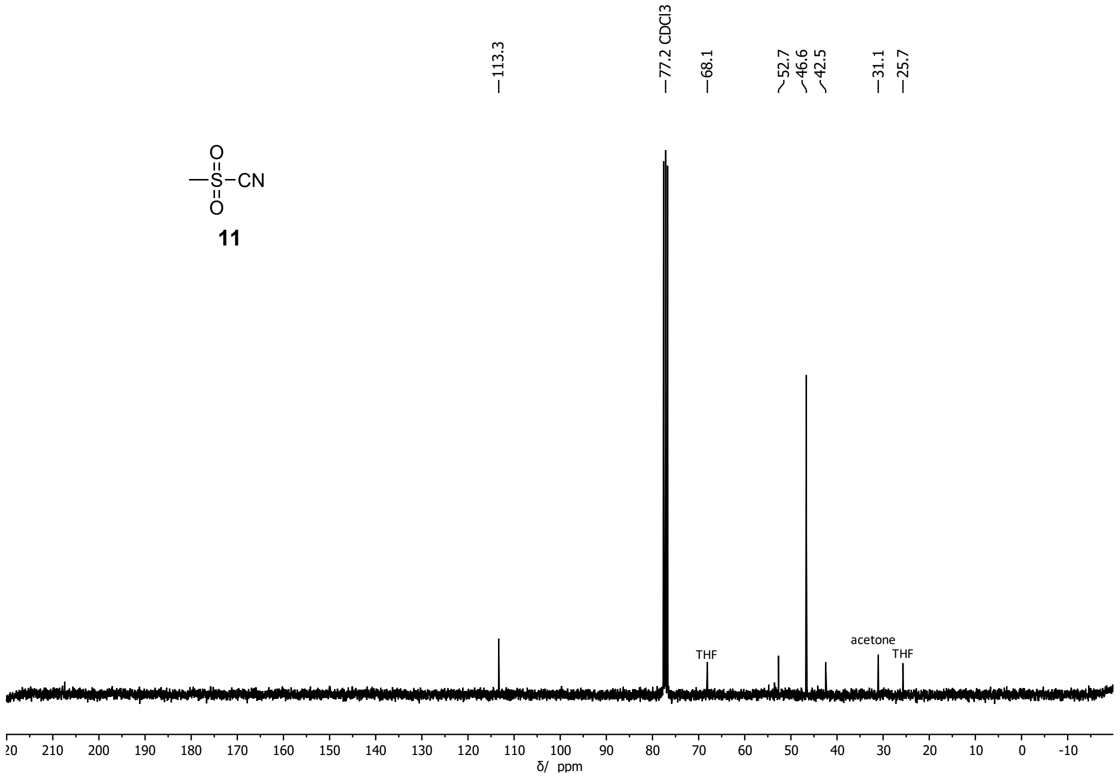

Figure S11: $^{13}C\{^{1}H\}$ NMR (75 MHz, $CDCl_3$) spectrum of methanesulfonyl cyanide (**11**).

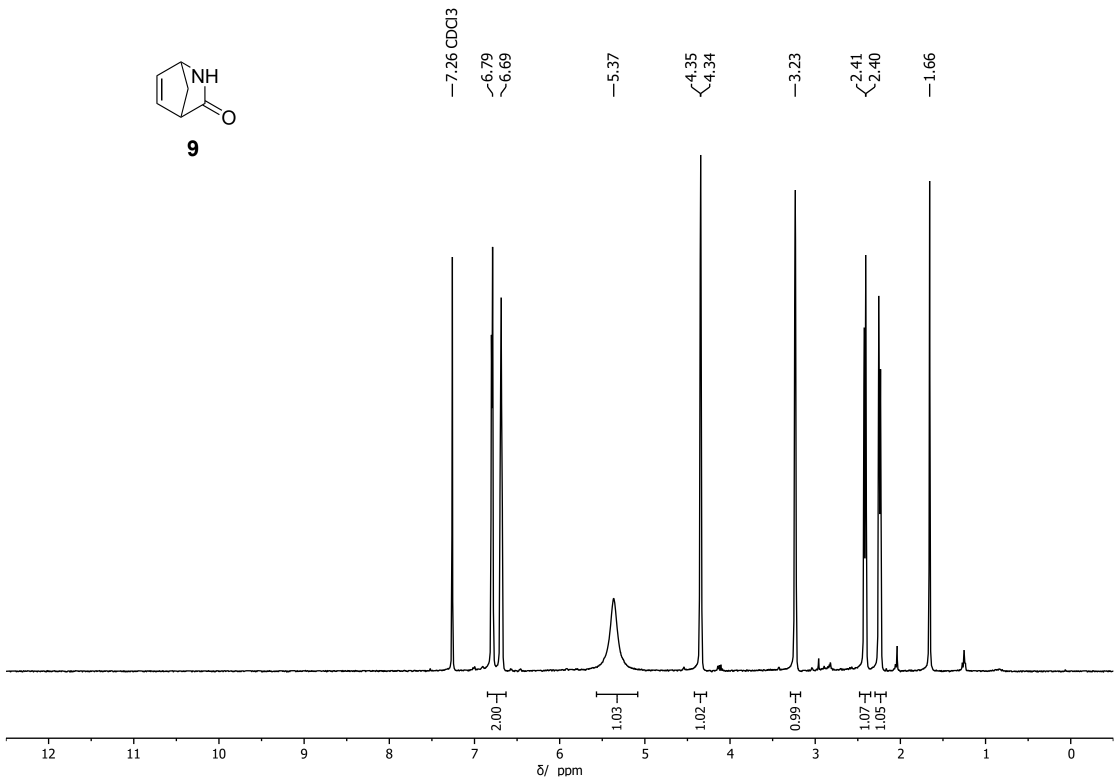

Figure S12: ^{1}H NMR (300 MHz, $CDCl_3$) spectrum of 2-azabicyclo[2.2.1]hept-5-en-3-one (Vince lactam, **9**).

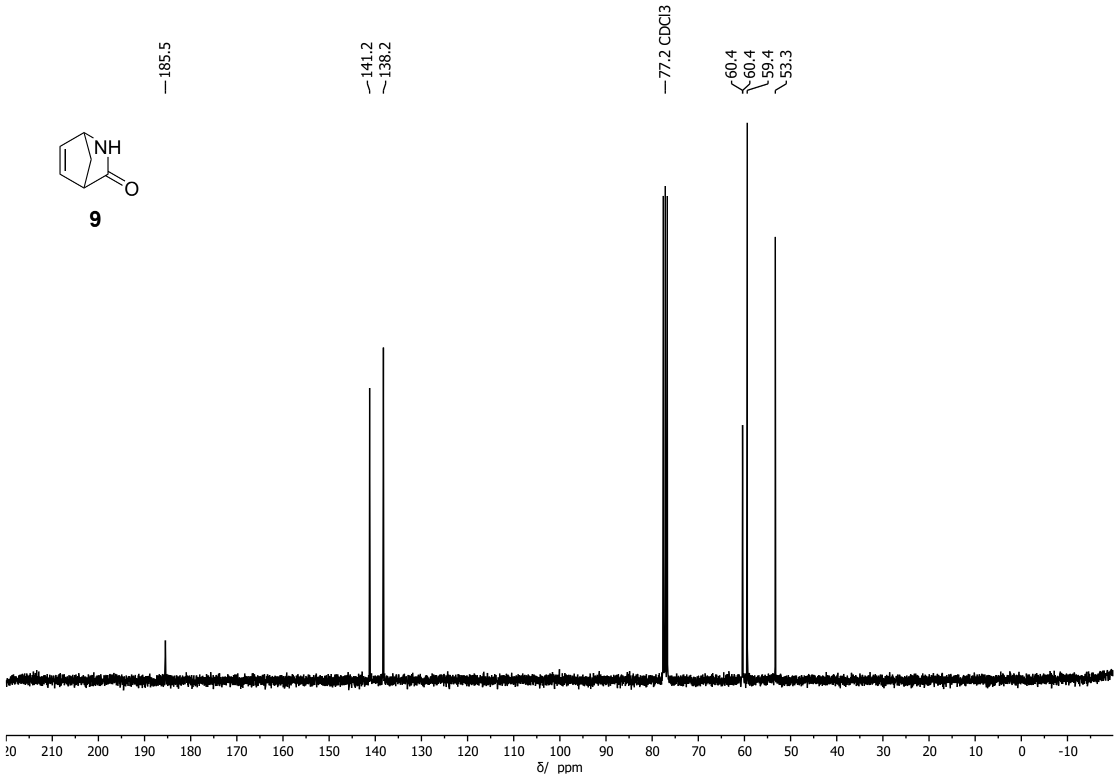

Figure S13: $^{13}C\{^{1}H\}$ NMR (75 MHz, $CDCl_3$) spectrum of 2-azabicyclo[2.2.1]hept-5-en-3-one (Vince lactam, **9**).

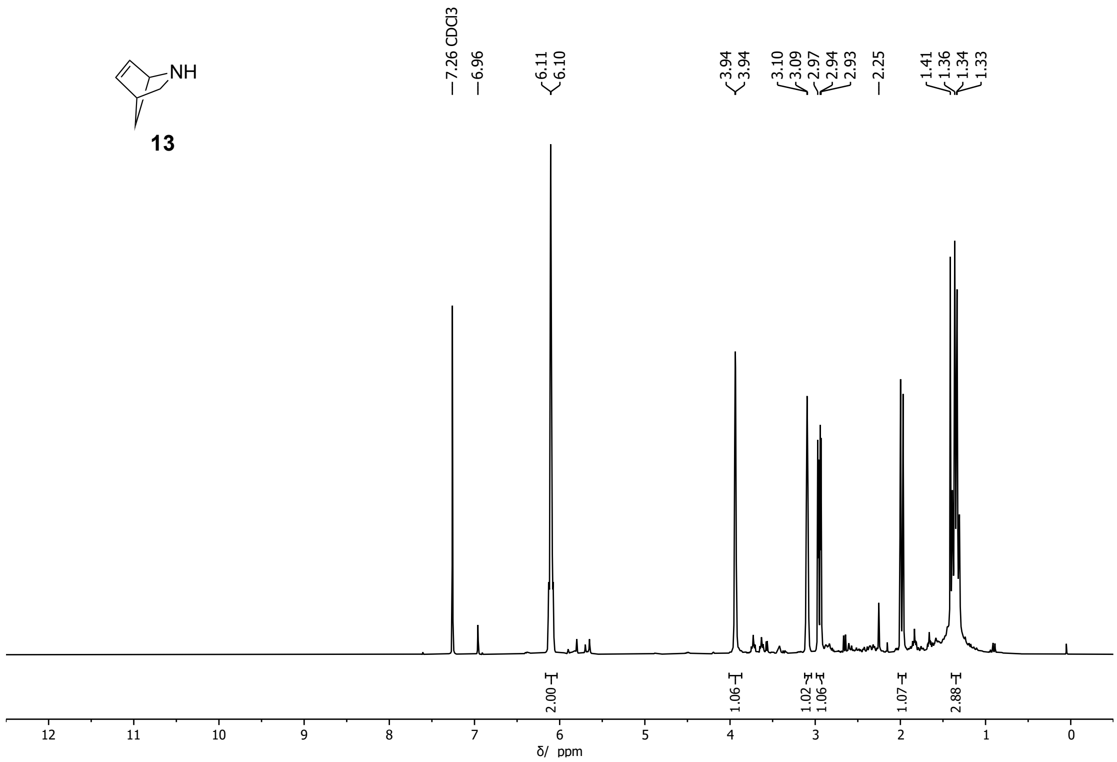

Figure S14: ^{1}H NMR (300 MHz, $CDCl_3$) spectrum of 2-azabicyclo[2.2.1]hept-5-ene (**13**).

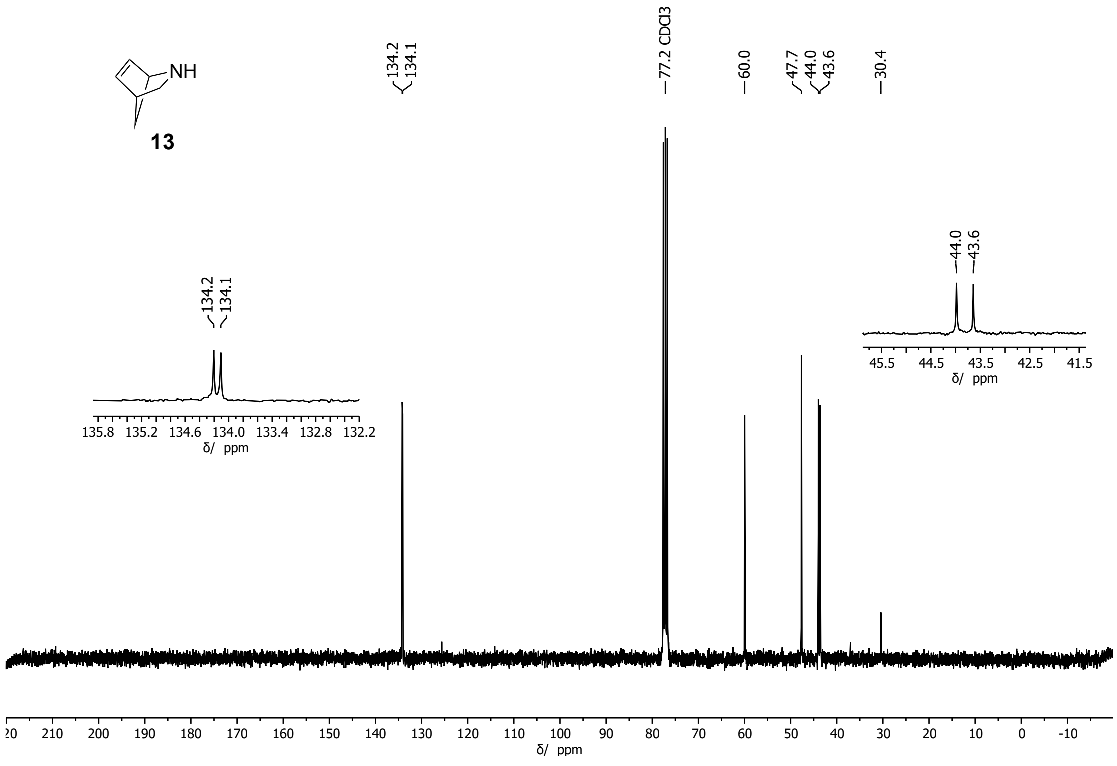

Figure S15: $^{13}C\{^1H\}$ NMR (75 MHz, $CDCl_3$) spectrum of 2-azabicyclo[2.2.1]hept-5-ene (**13**).

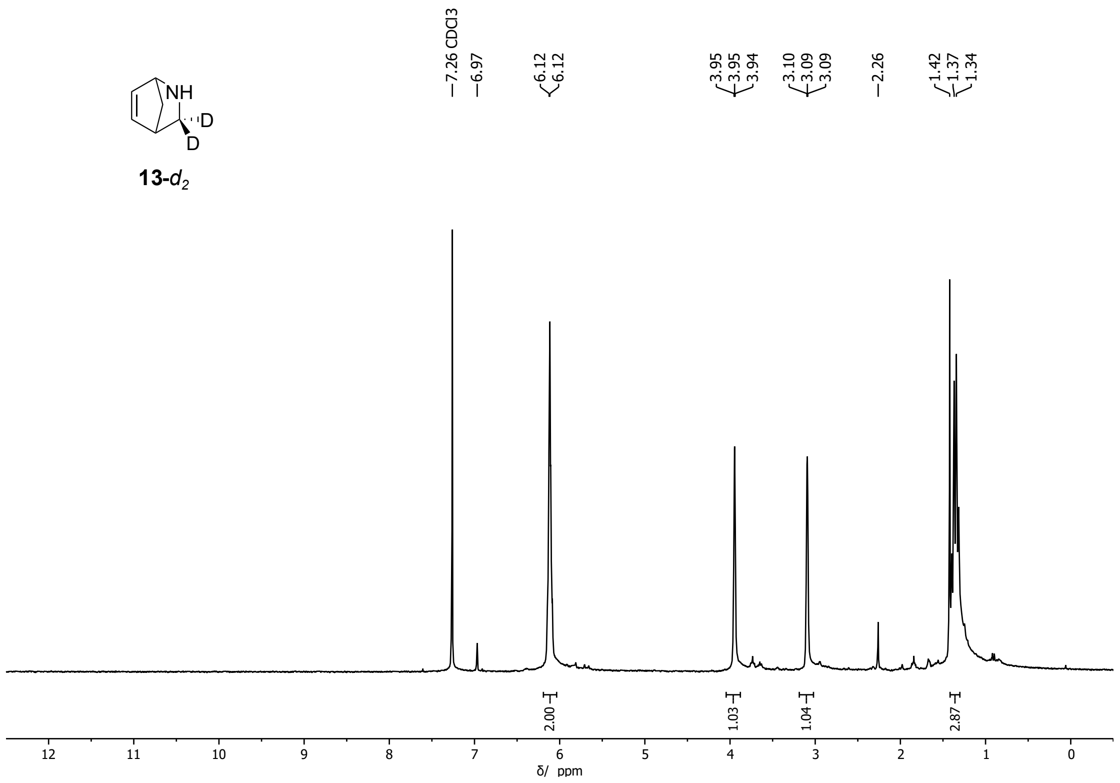

Figure S16: ^{1}H NMR (300 MHz, $CDCl_3$) spectrum of 2-azabicyclo[2.2.1]hept-5-ene-d_2 (**13**-d_2).

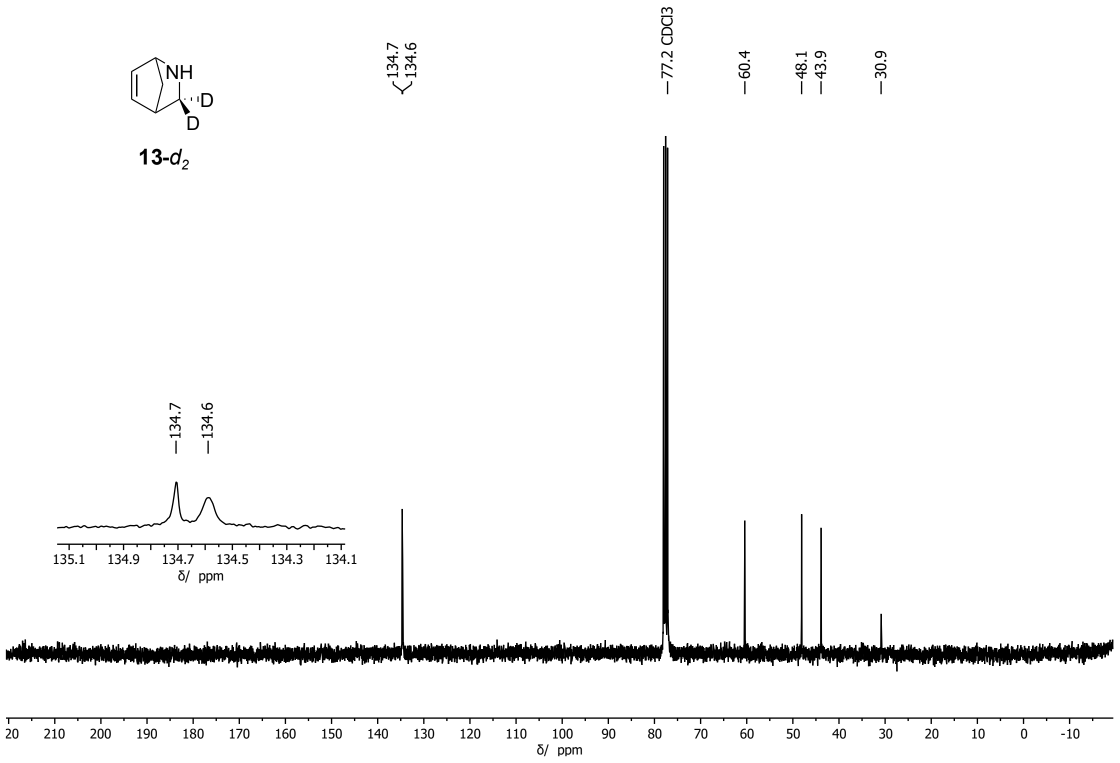

Figure S17: ^{13}C{^{1}H} NMR (75 MHz, $CDCl_3$) spectrum of 2-azabicyclo[2.2.1]hept-5-ene-d_2 (**13**-d_2).

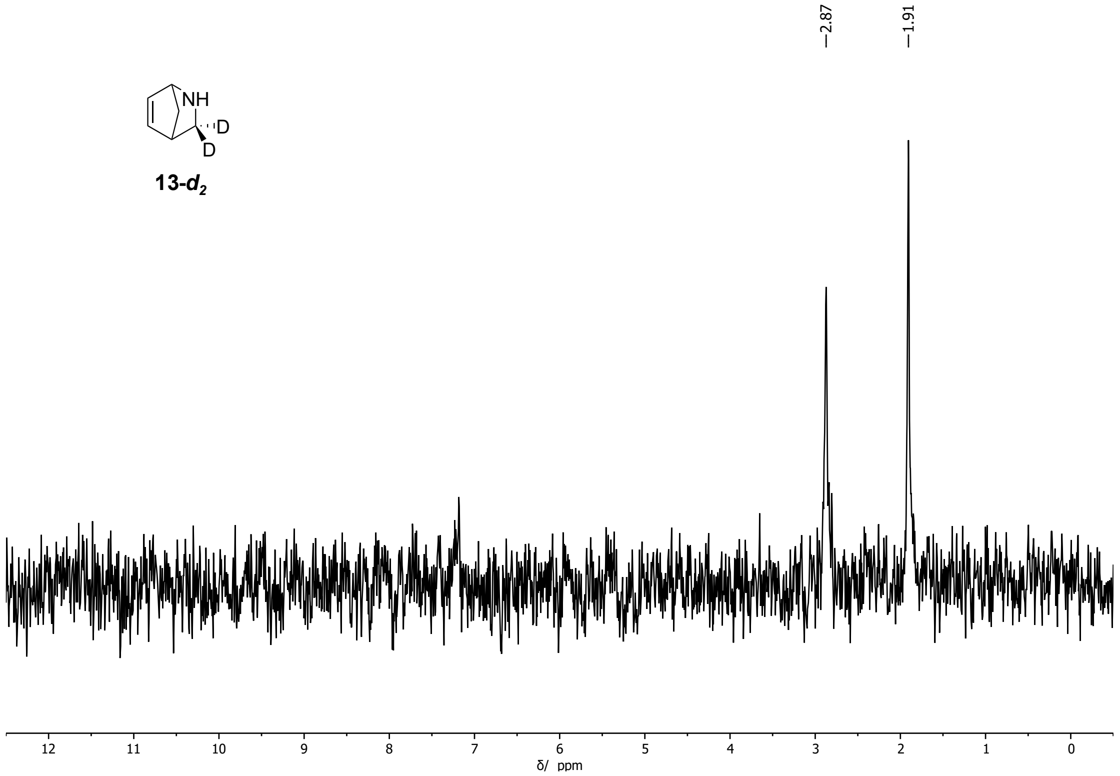

Figure S18: ^{2}H NMR (61 MHz, $CDCl_3$) spectrum of 2-azabicyclo[2.2.1]hept-5-ene-d2 (**13**-d_2).

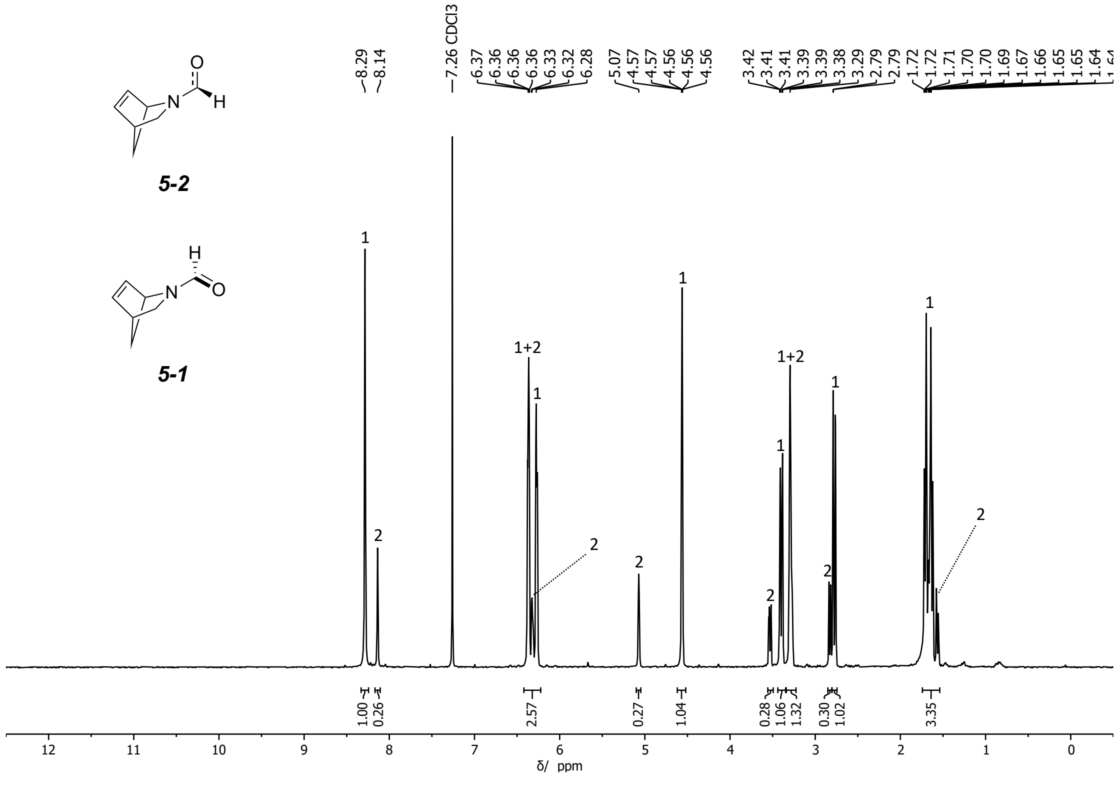

Figure S19: ^{1}H NMR (400 MHz, $CDCl_3$) of *N*-formyl-2-azabicyclo[2.2.1]hept-5-ene (**5**).

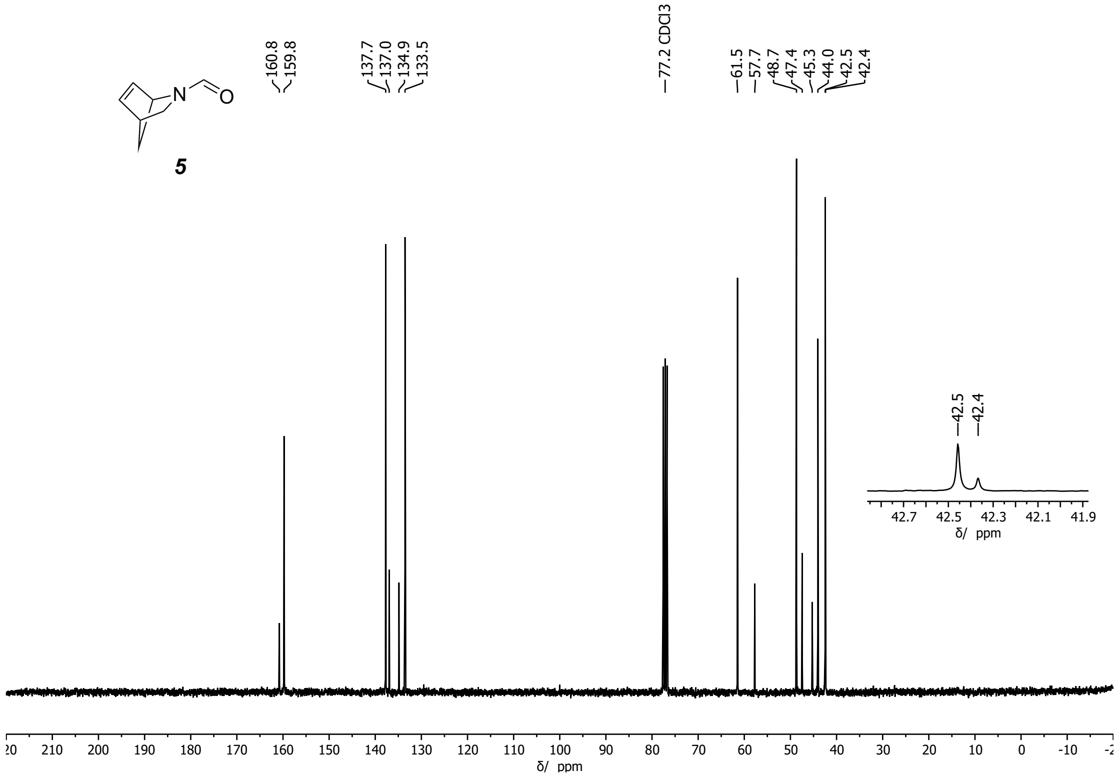

Figure S20: $^{13}C\{^{1}H\}$ NMR (75 MHz, $CDCl_3$) spectrum of *N*-formyl-2-azabicyclo[2.2.1]hept-5-ene (**5**).

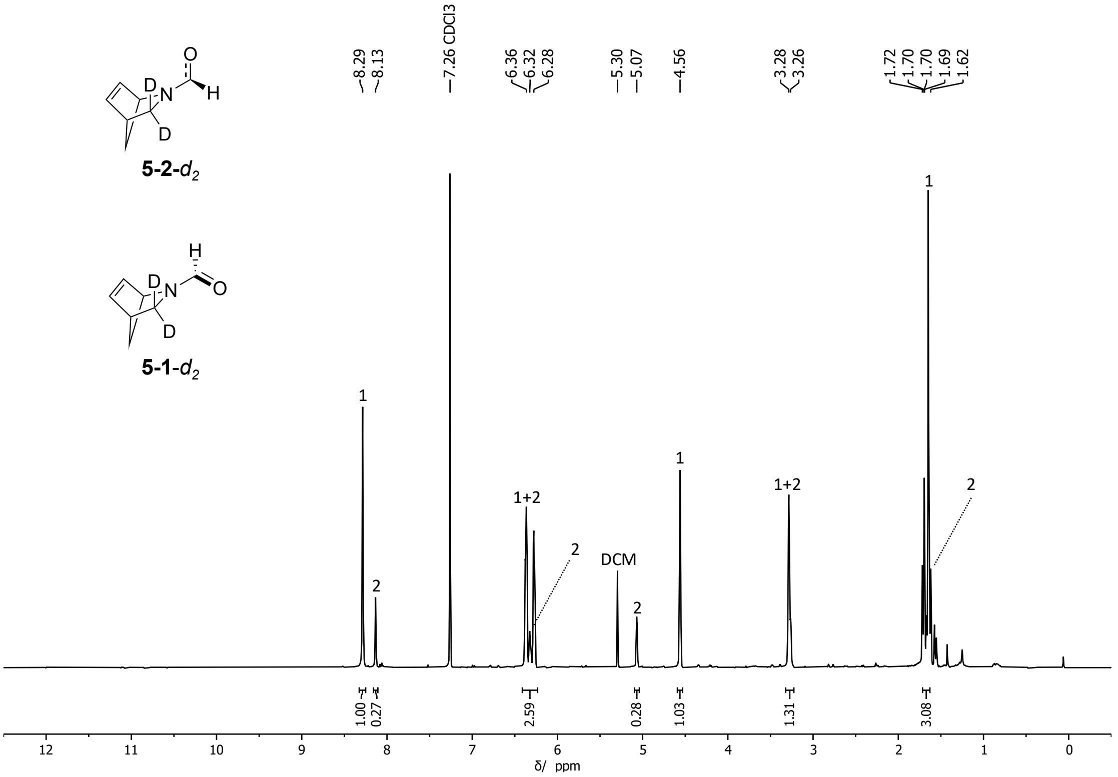

Figure S21: ^{1}H NMR (400 MHz, $CDCl_3$) spectrum of *N*-formyl-2-azabicyclo[2.2.1]hept-5-ene-d_2 (**5**-d_2). The peak positions are identical to the non-deuterated analog except the two pair of peaks at 3.4 ppm and 2.8 ppm, as well as 3.5 and 2.9 ppm are missing due to the deuterium substitution (Figure S21) The missing peaks appear in the ^{2}H NMR spectrum (Figure S25).

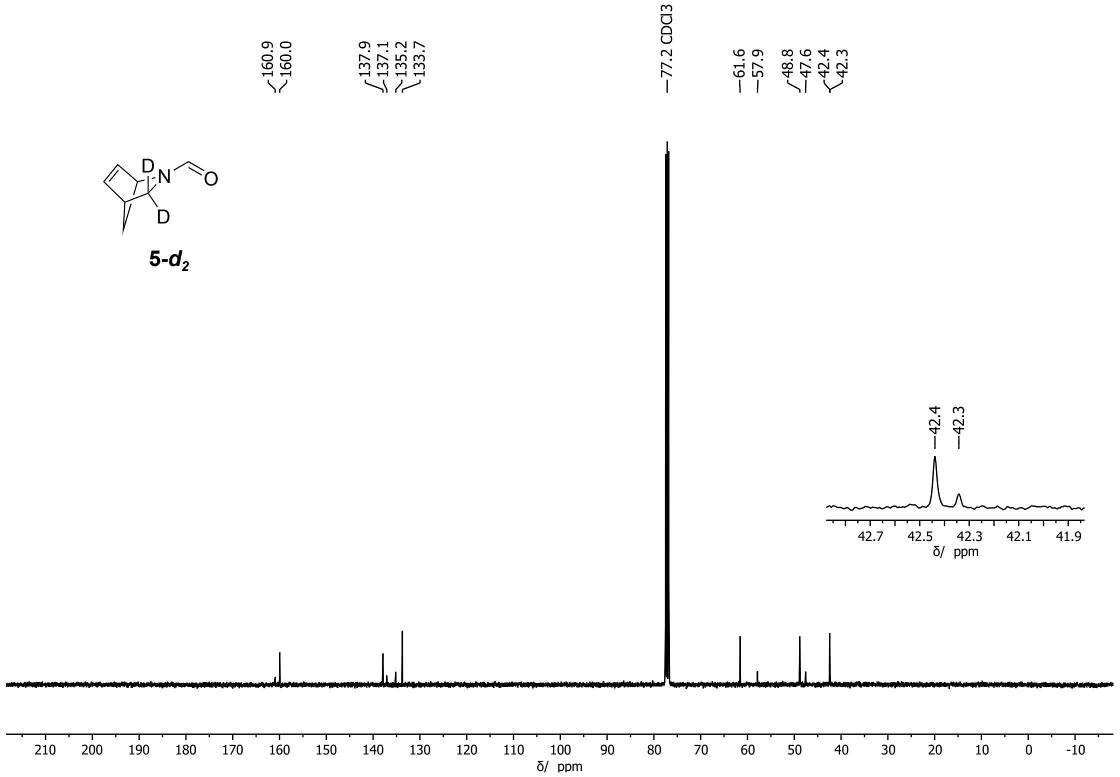

Figure S22: $^{13}C\{^{1}H\}$ NMR (101 MHz, $CDCl_3$) spectrum of *N*-formyl-2-azabicyclo[2.2.1]hept-5-ene-d_2 **(5-**d_2). The spectrum matches with the one of the non-deuterated analog (**5**, Figure S22) except the pair of peaks at 44.0 ppm and 43.5 ppm are not visible due to the deuterium substitution.

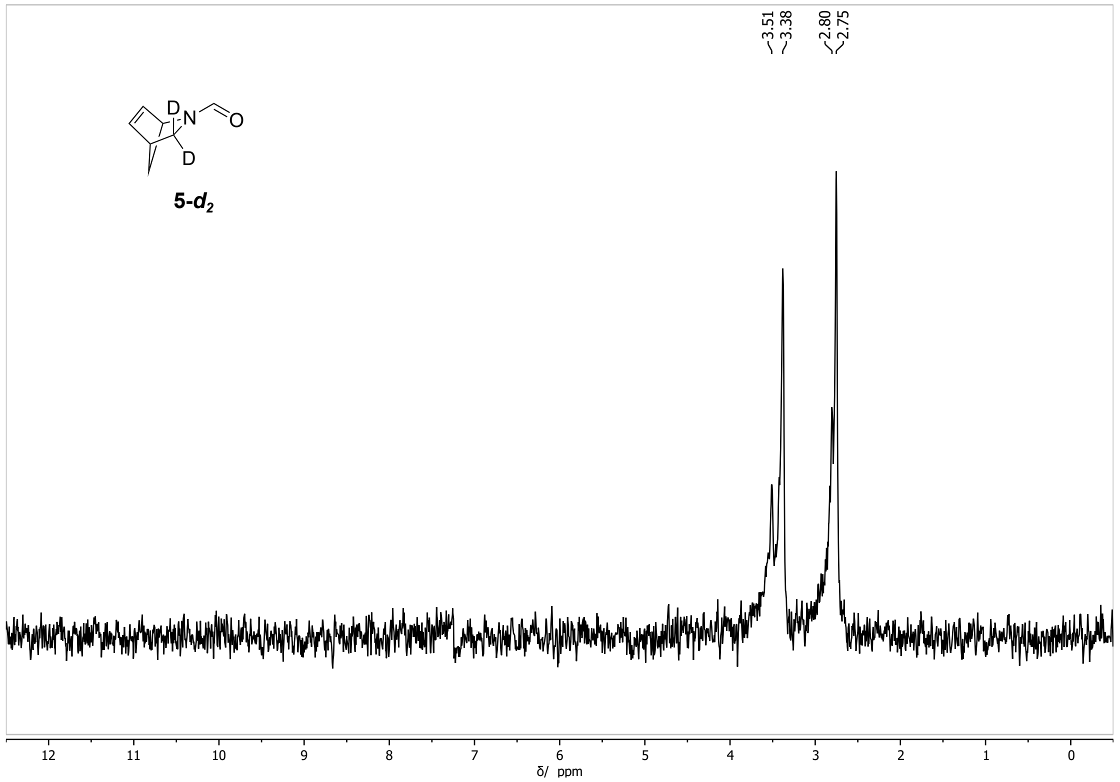

Figure S23: ^{2}H NMR (61 MHz, $CDCl_3$) spectrum of *N*-formyl-2-azabicyclo[2.2.1]hept-5-ene-d_2 (**5-**d_2).

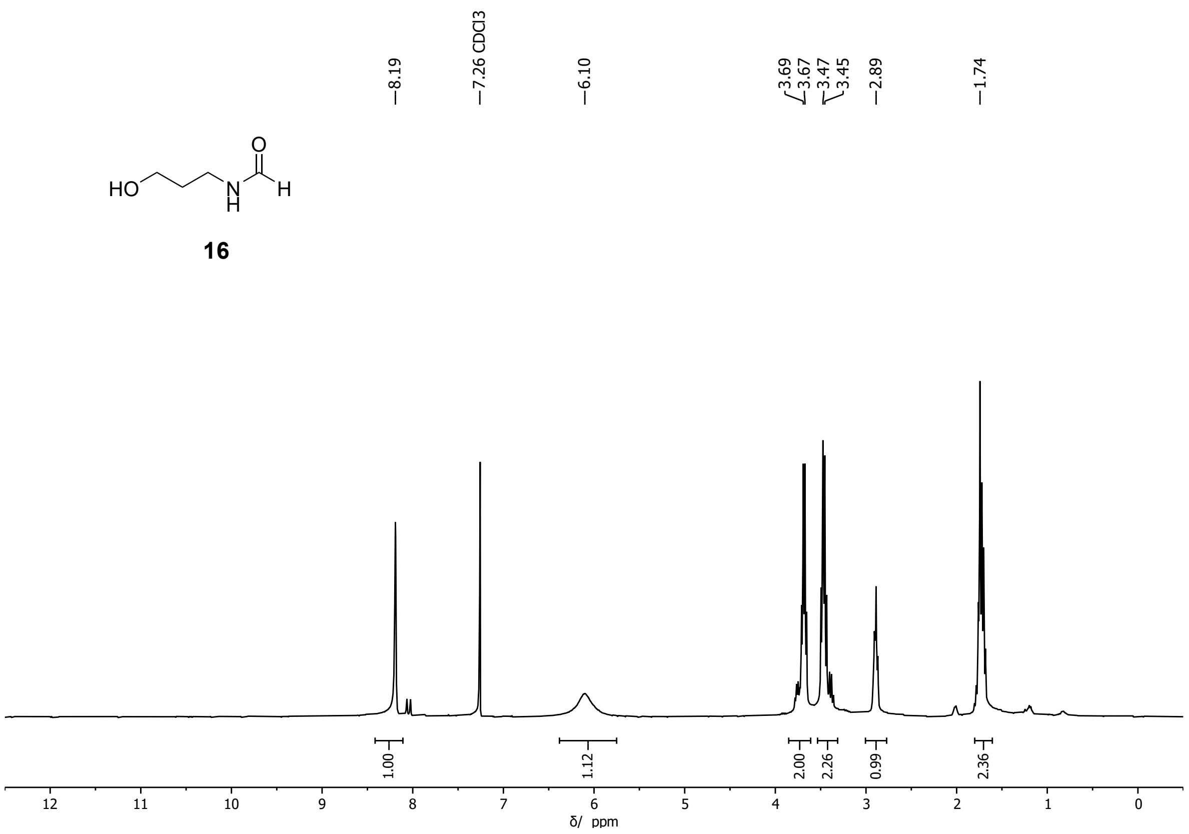

Figure S24: ^{1}H NMR (300 MHz, $CDCl_3$) spectrum of *N*-(3-hydroxypropyl)formamide (**16**).

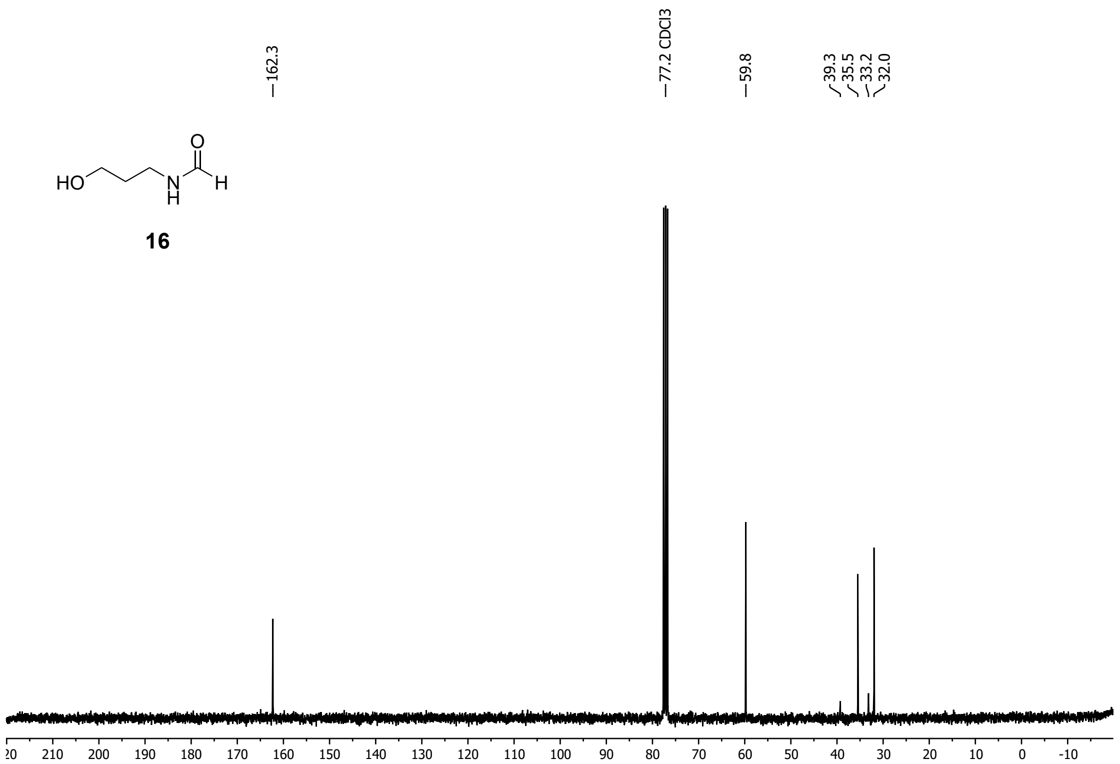

Figure S25: $^{13}C\{^{1}H\}$ NMR (101 MHz, $CDCl_3$) spectrum of *N*-(3-hydroxypropyl)formamide (**16**).

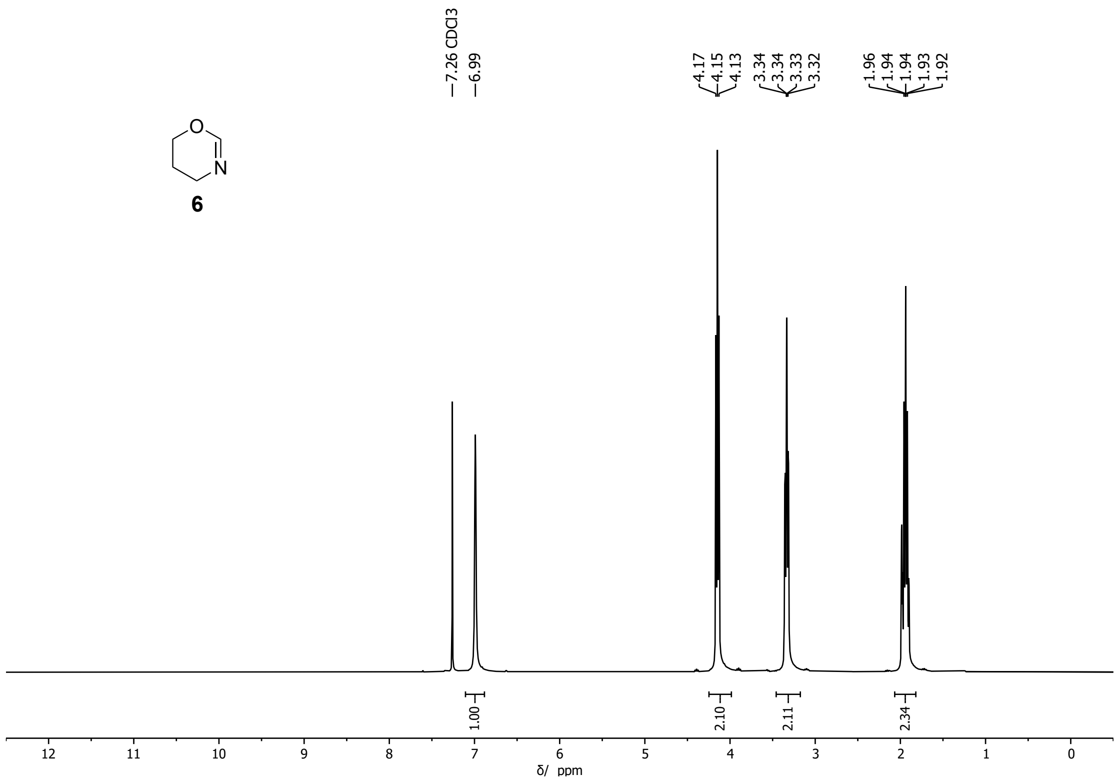

Figure S26: ^{1}H NMR (300 MHz, $CDCl_3$) spectrum of 5,6-dihydro-4H-1,3-oxazine (**6**).

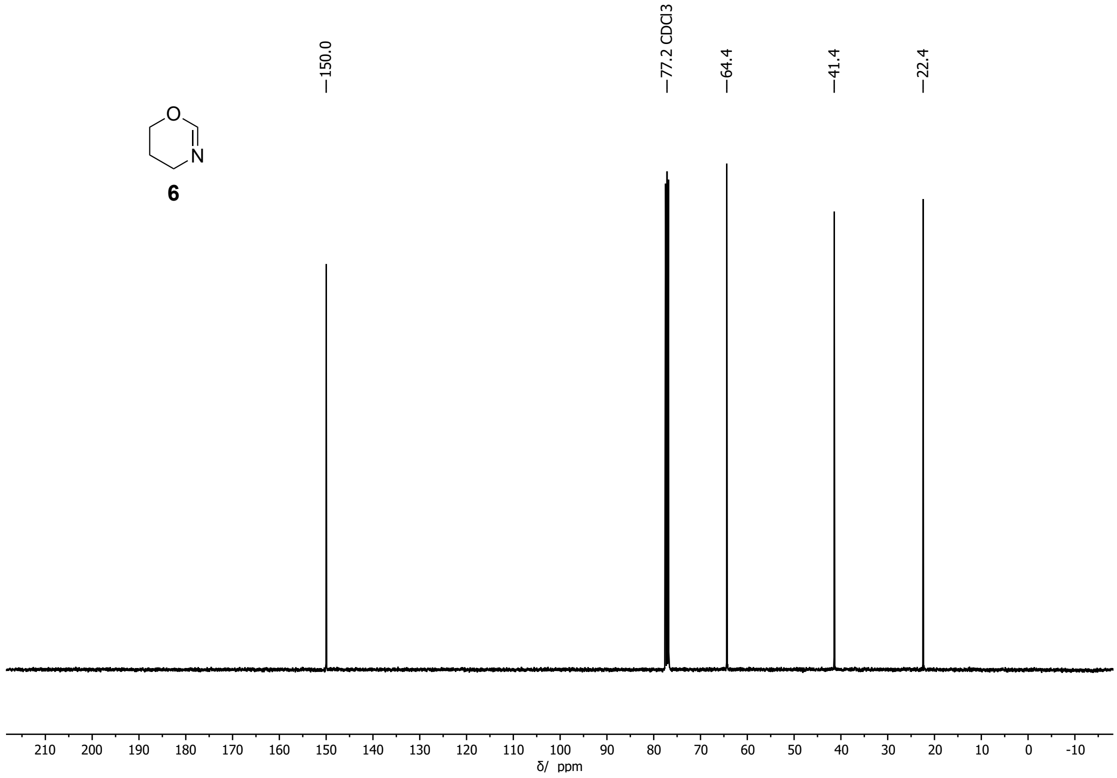

Figure S27: $^{13}C\{^{1}H\}$ NMR (101 MHz, $CDCl_3$) spectrum of 5,6-dihydro-4H-1,3-oxazine (**6**).

Potential Energy Surfaces

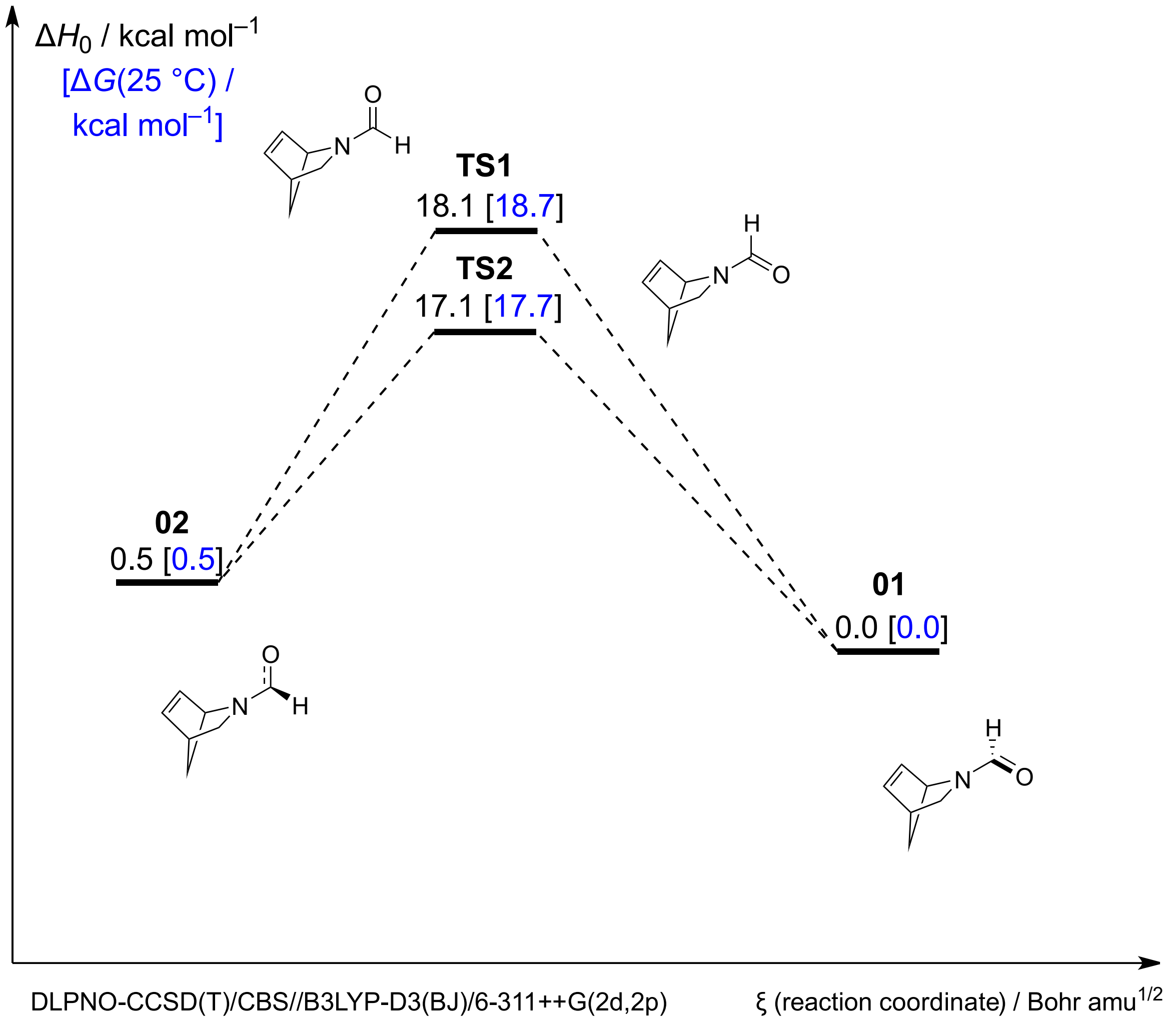

Figure S28: Potential energy surface (PES) around two conformers of *N*-formyl-2-azabicyclo[2.2.1]hept-5-ene (**5**).

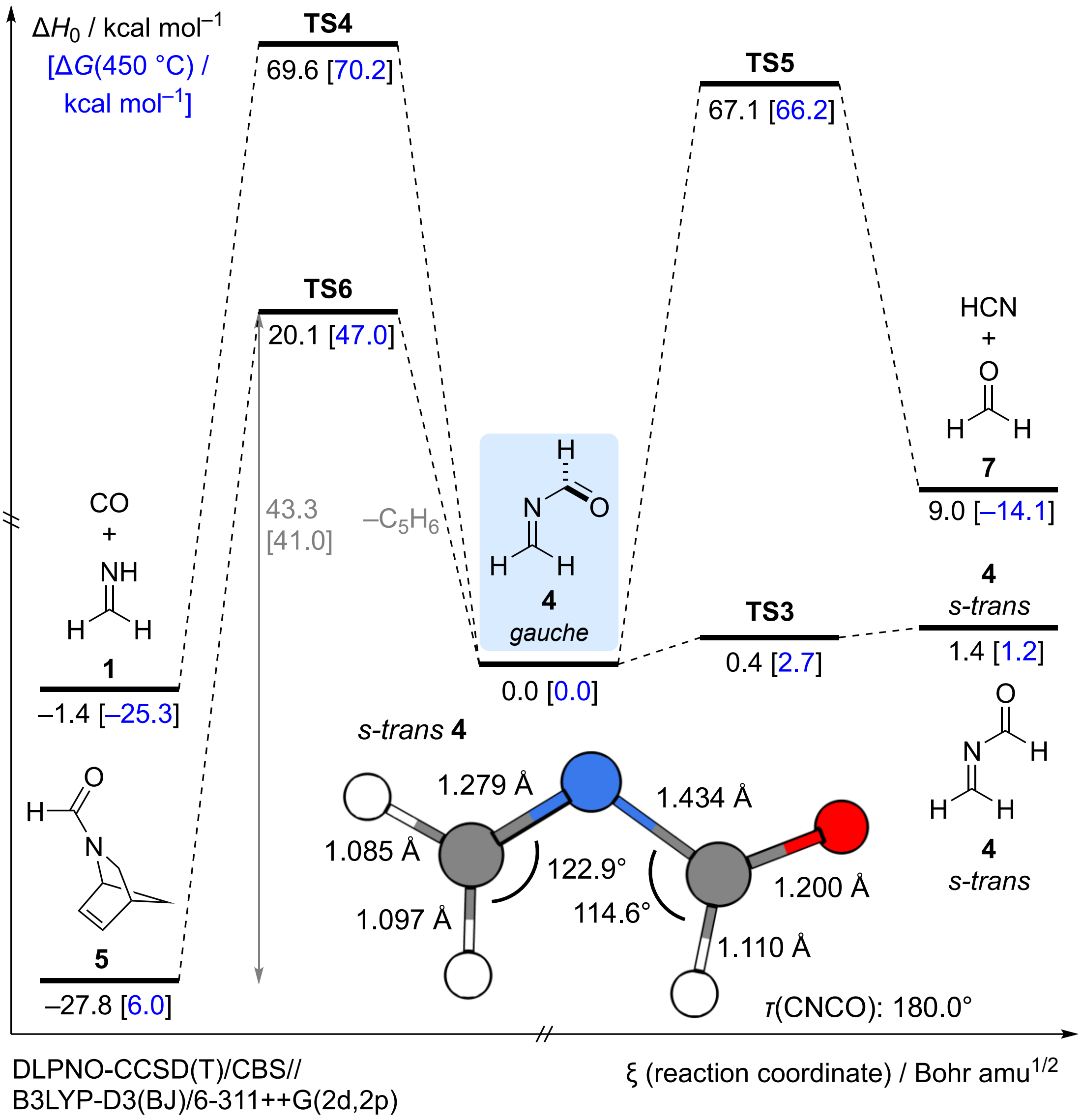

Figure S29: Alternative representation of Figure 4 with *N*-formyl-2-azabicyclo[2.2.1]hept-5-ene (**5**) as the starting material in the bottom left corner.

References

1 E. Whittle, D. A. Dows and G. C. Pimentel, *J. Chem. Phys.*, 1954, **22**, 1943.
2 L. Tauhardt, K. Kempe and U. S. Schubert, *J. Polym. Sci. Part Polym. Chem.*, 2012, **50**, 4516–4523.
3 M. C. Lasne, J. L. Ripoll and A. Thuillier, *J. Chem. Res. Synop.*, 1982, **8**, 214–215.
4 M. J. Frisch, G. W. Trucks, H. B. Schlegel, G. E. Scuseria, M. A. Robb, J. R. Cheeseman, G. Scalmani, V. Barone, G. A. Petersson, H. Nakatsuji, X. Li, M. Caricato, A. V. Marenich, J. Bloino, B. G. Janesko, R. Gomperts, B. Mennucci, H. P. Hratchian, J. V. Ortiz, A. F. Izmaylov, J. L. Sonnenberg, Williams, F. Ding, F. Lipparini, F. Egidi, J. Goings, B. Peng, A. Petrone, T. Henderson, D. Ranasinghe, V. G. Zakrzewski, J. Gao, N. Rega, G. Zheng, W. Liang, M. Hada, M. Ehara, K. Toyota, R. Fukuda, J. Hasegawa, M. Ishida, T. Nakajima, Y. Honda, O. Kitao, H. Nakai, T. Vreven, K. Throssell, J. A. Montgomery Jr., J. E. Peralta, F. Ogliaro, M. J. Bearpark, J. J. Heyd, E. N. Brothers, K. N. Kudin, V. N. Staroverov, T. A. Keith, R. Kobayashi, J. Normand, K. Raghavachari, A. P. Rendell, J. C. Burant, S. S. Iyengar, J. Tomasi, M. Cossi, J. M. Millam, M. Klene, C. Adamo, R. Cammi, J. W. Ochterski, R. L. Martin, K. Morokuma, O. Farkas, J. B. Foresman and D. J. Fox, Gaussian 16 Rev. C.01 2016.
5 A. D. Becke, *Phys. Rev. A*, 1988, **38**, 3098–3100.
6 A. D. Becke, *J. Chem. Phys.*, 1993, **98**, 5648–5652.
7 C. Lee, W. Yang and R. G. Parr, *Phys. Rev. B*, 1988, **37**, 785–789.
8 R. Krishnan, J. S. Binkley, R. Seeger and J. A. Pople, *J. Chem. Phys.*, 1980, **72**, 650–654.
9 T. Clark, J. Chandrasekhar, G. W. Spitznagel and P. V. R. Schleyer, *J. Comput. Chem.*, 1983, **4**, 294–301.
10 S. Grimme, S. Ehrlich and L. Goerigk, *J. Comput. Chem.*, 2011, **32**, 1456–1465.
11 Chemcraft - graphical software for visualization of quantum chemistry computations. Version 1.8, build 654. https://www.chemcraftprog.com.
12 F. Neese, *WIREs Comput. Mol. Sci.*, 2022, **12**, e1606.
13 D. A. Matthews, L. Cheng, M. E. Harding, F. Lipparini, S. Stopkowicz, T.-C. Jagau, P. G. Szalay, J. Gauss and J. F. Stanton, *J. Chem. Phys.*, 2020, **152**, 214108.
14 S. Grimme, *J. Chem. Phys.*, 2006, **124**, 034108.
15 J.-D. Chai and M. Head-Gordon, *Phys. Chem. Chem. Phys.*, 2008, **10**, 6615–6620.
16 V. Pirenne, G. Kurtay, S. Voci, L. Bouffier, N. Sojic, F. Robert, D. M. Bassani and Y. Landais, *Org. Lett.*, 2018, **20**, 4521–4525.
17 G. J. Griffiths and F. E. Previdoli, *J. Org. Chem.*, 1993, **58**, 6129–6131.
18 A. Heesing and W. Herdering, *Chem. Ber.*, 1983, **116**, 1081–1096.
